# Tools for Gene-Regulatory Analyses in the Marine Annelid *Platynereis dumerilii*

**DOI:** 10.1371/journal.pone.0093076

**Published:** 2014-04-08

**Authors:** Benjamin Backfisch, Vitaly V. Kozin, Stephan Kirchmaier, Kristin Tessmar-Raible, Florian Raible

**Affiliations:** 1 Max Ferdinand Perutz Laboratories (MFPL), University of Vienna, Vienna, Austria; 2 Research Platform “Marine Rhythms of Life,” University of Vienna, Vienna, Austria; 3 Department of Embryology, St. Petersburg State University, St. Petersburg, Russia; Institute of Molecular and Cell Biology, Singapore

## Abstract

The advent of high-throughput sequencing technology facilitates the exploration of a variety of reference species outside the few established molecular genetic model systems. Bioinformatic and gene expression analyses provide new ways for comparative analyses between species, for instance, in the field of evolution and development. Despite these advances, a critical bottleneck for the exploration of new model species remains the establishment of functional tools, such as the ability to experimentally express genes in specific cells of an organism. We recently established a first transgenic strain of the annelid *Platynereis*, using a Tc1/mariner-type Mos1 transposon vector. Here, we compare Mos1 with Tol2, a member of the hAT family of transposons. In *Platynereis*, Tol2-based constructs showed a higher frequency of nuclear genome insertion and sustained gene expression in the G0 generation. However, in contrast to Mos1-mediated transgenes, Tol2-mediated insertions failed to retain fluorescence in the G1 generation, suggesting a germ line-based silencing mechanism. Furthermore, we present three novel expression constructs that were generated by a simple fusion-PCR approach and allow either ubiquitous or cell-specific expression of a reporter gene. Our study indicates the versatility of Tol2 for transient transgenesis, and provides a template for transgenesis work in other emerging reference species.

## Introduction

The selection of a few species as “molecular model systems” has helped researchers to gain deep insight into the molecular processes underlying cell biology and development of these organisms. The wealth of data and the power of molecular tools available for these species strongly contrast with the experimental amenability of most other organisms for molecular investigation, even where such organisms exhibit biological phenomena not covered by the standard models. It has therefore been argued that biologists need to start selecting and exploring new model systems [1], [2].

One step into this direction has been enabled by the development of cost-effective massive sequencing technology. This technology has facilitated the deciphering of an increasing number of genomes and transcriptomes of potential new model systems. More than 150 eukaryotic genomes and an even greater number of transcriptomes already provide a valuable resource for phylogenetic or comparative genomic analyses (see e.g. refs. [3], [4], [5], [6]). Besides their value for bioinformatics approaches, such sequence resources also provide gene sets for expression analyses, thereby allowing insight into the dynamics and/or spatial specificity of transcription during development. Systematic gene expression data have been generated for conventional model species (see e.g. refs. [7], [8]), and also serve as a basis for the comparison of gene expression patterns with and between emerging model species, thereby providing insight into the evolution of tissues and cell types (reviewed in ref. [9]).

Despite these advances, the functional amenability of many emerging model systems is still limited. One limitation concerns the capacity of new model species to generate insight into gene or cell functions that may differ from those functions established in conventional model species. This is especially relevant where new model species are phyletically distant from well-studied models, and significantly differ in their genetic repertoire. Likewise, this limitation also applies where new models display distinct biological traits that can simply not be studied in traditional systems. In such cases, it is necessary to develop, along with genomic and transcriptomic resources, also tools that allow to use these resources for functional studies in the respective organism itself.

DNA transposons are mobile genetic elements that can transport DNA inserts of varying length. Their mobilization typically relies on the presence of an enzyme (transposase) acting on a characteristic flanking DNA sequence containing transposon-specific inverted repeats. Transposon-based transgenesis forms the basis of various functional approaches, ranging from targeted expression of candidate genes or functional reporters to insertional mutagenesis (reviewed in ref. [10]). The establishment of transgenic technology has therefore strongly contributed to the transition of other species to molecular model systems, for instance the ascidian *Ciona intestinalis* [11], [12]. Moreover, whereas more widely used techniques like immunohistochemistry, whole-mount in situ hybridization or transcriptomic approaches only provide “snapshots” of development, transgenesis with fluorescent reporter constructs allows continuous observation of organelles and cells, and specific manipulation also at later stages of development.

Lophotrochozoans constitute a large superphylum of protostome animals that are distinct from the ecdysozoans [13], [14]. Ecdysozoans include species like the nematode *Caenorhabditis elegans* or the fruitfly *Drosophila melanogaster* that are well-established functional model systems with a large panel of functional tools. Several lophotrochozoans have become prominent developmental model systems, including leeches and planarians, but given the large number of species in this superphylum, and the relevance of lophotrochozoan groups such as mollusks and annelids for marine biology, the amount of molecular data for lophotrochozoans is rather sparse, with the first genomes just becoming available [6], [15], [16].

Among the lophotrochozoans, *Platynereis dumerilii* has gained relevance as an annelid reference species for eye and brain development and evolution (see e.g. refs. [17], [18], [19]). Strains for *Platynereis* can be easily bred in the lab, and various molecular resources and techniques facilitate analyses of its cell types and tissues, and their comparison with other animal models [18], [20], [21], [22]. Whereas reports on transgenic lophotrochozoans were previously limited to planarians [23] and platyhelminths [24], we have recently obtained a first transgenic *Platynereis* strain, after microinjecting zygotes with a Tc1/*mariner*-type vector harboring an r-opsin::egfp reporter construct [25].

Despite this positive example, several fundamental aspects of transgenesis in *Platynereis* have so far not been addressed. This includes the question if the delivered constructs are excised from donor DNA and inserted into the host genome, or maintained by other mechanisms such as epichromosomal arrays. The mode of transmission is expected to impact on the stability of transgene expression throughout subsequent generations. Moreover, there is currently no estimate of the efficiency of Mos1-mediated transgenesis, and how this compares to the efficiency of other classes of transposons in *Platynereis*. Such estimates will be important for deciding on the best transgenesis strategy to explore for future and larger-scale experiments.

Here, we present a detailed analysis of transposase-mediated transgenesis in *Platynereis*. We provide evidence that reporters are cleaved in a transposase-dependent fashion, and inserted in the host genome. To address if there are differences between the applicability of different transposon classes in *Platynereis* transgenesis, we compared the Mos1 system with Tol2, a member of the hAT family of transposons used in a wide variety of model systems. Whereas we find Tol2 to be around threefold more efficient for transient transgenesis than Mos1, Tol2-mediated insertions appeared to be silenced in the germ line. This is in contrast to Mos1, for which we were able to generate another stable transgenic strain. Assays for Mos1-mediated excision and a study of integration sites suggests that in the bristle worm, Mos1 transposition is impaired in its efficiency, and accompanied by non-homologous repair. Our data indicate that each of the two delivery systems has individual advantages for *Platynereis* transgenesis, and that transient transgenesis efficiencies are not necessarily a suitable proxy for efficiencies of stable germline transgenesis.

## Results

### Cleavage and Excision of Transposon-based Reporter Constructs Delivered by Microinjection

To maximize compatibility between the Mos1 and Tol2 systems, we used re-engineered target vectors of both transposon systems that carried the same restriction sites. We chose a head-to-head arrangement of two I-SceI target sites. As the I-SceI target site is 18 nucleotides long, it will theoretically occur every 7×10^10^ nucleotides, or once per 70 haploid *Platynereis* genomes, using the recent genome size estimate of 1 Gbp [26]. We therefore expect that the vectors we generated should be compatible with cloning of most or all desirable genomic fragments of *Platynereis*, as well as many other species, making these vectors a useful resource for the community. The I-SceI recognition sites can be easily inserted into flanking primers designed to amplify a given expression construct. Furthermore, if needed, reporters can also be released by co-injected I-SceI meganuclease. In *Platynereis*, meganuclease has so far only led to mosaic expression of a tested reporter (Kristin Tessmar-Raible, unpublished), but in fishes, amphibians, ascidians, or cnidarians, meganuclease-mediated transgenesis is an alternative to transposon-based approaches (see refs. [27] and [28] and references therein). As exemplified in this study, simple expression constructs can be generated by fusion PCR, a technique also used for the generation of nematode expression constructs [29]. For more complex regulatory regions, an alternative is the recombineering of larger genomic clones to insert a reporter cassette, followed by transfer of the recombineered locus into the target vector [25], [30].

**Figure 1:**
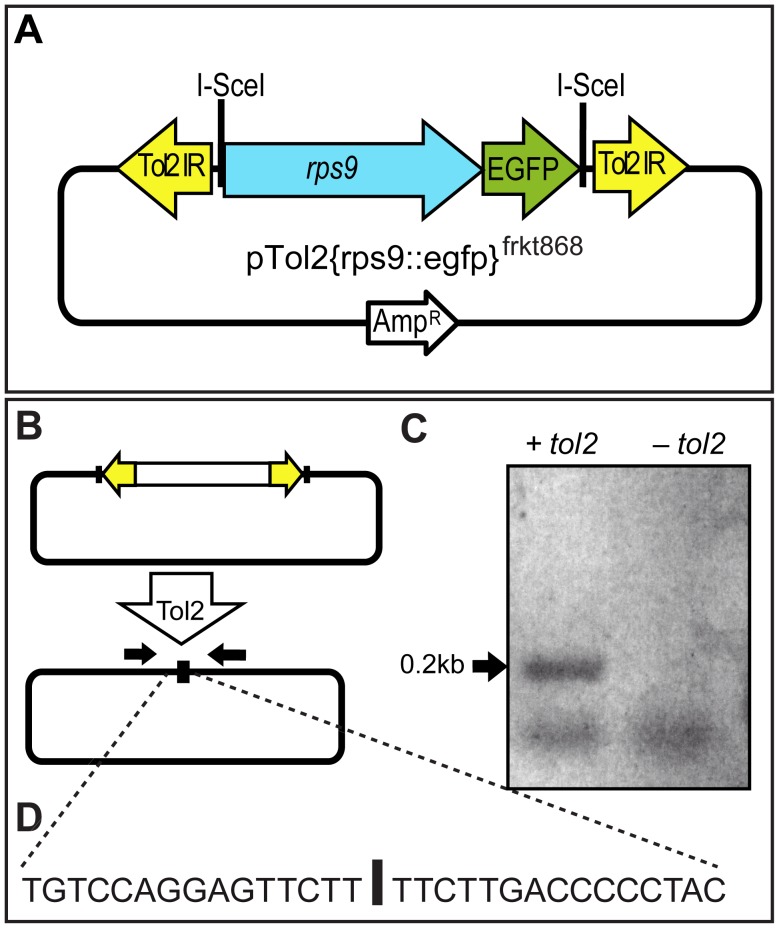
Excision of a microinjected Tol2-based construct. (**A**) Scheme of donor vector pTol2{rps9::egfp}^frkt868^; the rps::egfp cassette is flanked by inverted Tol2 repeats (Tol2IR) that serve as recognition site for Tol2 transposase co-injected as mRNA along with the vector DNA. (**B, C**) PCR using vector-specific primers (black arrows) yields a 200 bp PCR fragment specifically from embryos co-injected with *tol2* transposase mRNA (left lane), but not from controls (right lane). (**D**) Precise cleavage of the reporter construct at the end of the Tol2 IRs is evidenced by sequencing of the 200 bp fragment.

**Figure 2:**
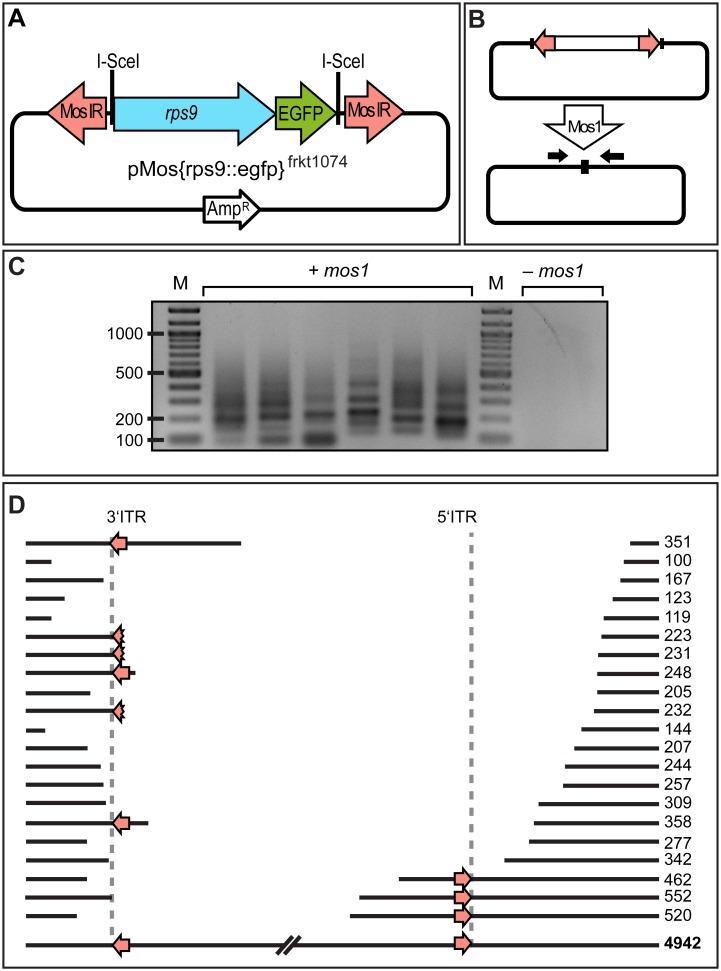
Excision of a microinjected Mos-based construct. (**A**) Scheme of donor vector pMos{rps9::egfp}^frkt1074^; in analogy to pTol2{rps9::egfp}^frkt868^ (Figure 1A), the rps::egfp cassette is flanked by inverted Mos repeats (MosIR) that serve as recognition site for Mos1 transposase. (**B, C**) PCR using vector-specific primers (black arrows) yields a variety PCR fragment from embryos co-injected with *mos1* transposase mRNA (left lanes), but not controls (right lanes). (**D**) Sequencing of 21 individual PCR product reveals imprecise breakpoints caused by impaired excision of reporter constructs and/or subsequent modification of DNA ends in the context of non-homologous end repair. See **Alignments S1** and **S2** for details.

For the determination of transgenesis efficiency, we decided to first generate a ubiquitous reporter construct. For this, we focused on *Platynereis rps9*, a gene encoding the ubiquitously expressed ribosomal protein Rps9. Using a thermal asymmetric interlaced polymerase chain reaction (TAIL-PCR) approach [11], [31], we isolated around 3 kB of DNA covering the 5′UTR and upstream sequence of the *rps9* gene. A 1.6 kB fragment of this piece was fused to the enhanced green fluorescent protein (*egfp*) gene by fusion PCR. This *rps9::egfp* construct was inserted into both the Tol2 and Mos target vector, yielding the constructs pTol2{rps9::egfp}^frkt868^ (**Figure 1A**) and pMos{rps9::egfp}^frkt1074^ (**Figure 2A**), respectively.

For delivering these constructs into animals, we microinjected freshly fertilized *Platynereis* zygotes. To monitor transposase activity in the living worms, we used a plasmid excision strategy that tests if the reporter construct flanked by transposon repeats is excised from the donor plasmid when co-injected with synthetic transposase mRNA [32]. We designed primers matching the flanking regions of the Tol2-based donor vector, outside of the inverted transposon repeats (**Figure 1B**). Upon release of the insert, these primers should amplify a distinct fragment of about 200 bp in an mRNA-dependent fashion. Indeed, only zygotes co-injected with *tol2* mRNA gave rise to a PCR amplicon of the expected size (**Figure 1C**). Subcloning and sequencing of this amplicon confirmed that it is identical to the expected donor fragment, and precisely lacks the inverted transposon repeats (**Figure 1D**). These results establish that no significant endogenous Tol2 transposase activity is present in *Platynereis* zygotes, and that injected *tol2* transposase mRNA yields sufficient enzyme activity to excise the reporter *in vivo* from the donor cassette during the first rounds of cell divisions.

Similar to the case of Tol2, co-injection of *mos1* transposase mRNA with Mos1 ITR-flanked rps9::egfp reporter, followed by PCR amplification with vector-specific primers, resulted in the recovery of shorter donor DNA amplicons (**Figure 2B, C**). However, in contrast to the case of Tol2, these assays yielded various fragment sizes, differing between injected individuals (**Figure 2C**). Consistent with this observation, sequencing of 21 amplicons resulting from Mos1 excision assays indicated various deletions along the vector and insert, both comprising and excluding the full ITR sequences (**Figure 2D** **, Alignments S1/S2**). Moreover, in 7 of the 21 amplicons, we detected additional small insertions between the respective breakpoints, varying in length between 1 and 24 nucleotides. The observed products appear incompatible with a regular Mos1-mediated excision, but rather resemble our observations for products generated by non-homologous end joining repair (see discussion). Even though the observed amplicons appeared to be irregular, their occurrence was dependent on co-injection of *mos1* mRNA (**Figure 2C**). This indicates that there is no endogenous transposase activity that can act on Mos1 ITRs in *Platynereis*, but hints at an impairment of efficient Mos1 excision and/or transposition in the worms.

### Robust Tol2-mediated Transgenesis in G_0_

**Figure 3:**
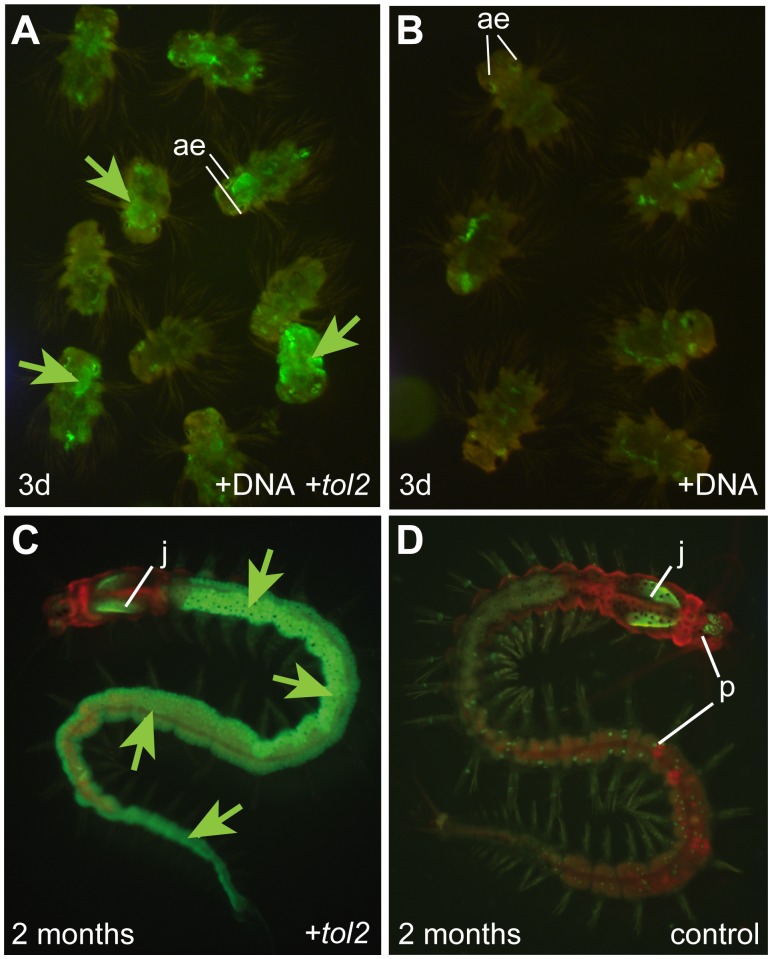
Tol2-mediated transgenesis with pTol2{rps9::egfp} yields robust, ubiquitous expression of EGFP in G_0_ animals. (**A, B**) pTol2{rps9::egfp}^frkt868^-injected animals co-injected with *tol2* mRNA (A) show significantly stronger EGFP fluorescence (green arrows) than those without transposase (B) (ae: autofluorescence around the adult eyes). (**C, D**) Stable ubiquitous EGFP expression (green arrows) two months after co-injection of donor DNA and *tol2* mRNA (C) compared to a non-injected animal (D). Labels indicate autofluorescence of the jaws (j) as well as the iridophore pigments (p) in both the head and trunk.

Next, we tested if Tol2 transposase also catalyzed stable genomic integration of the excised fragment. As a first indication, we compared fluorescence of animals 3 days after zygote injection. When we investigated EGFP fluorescence in larvae that received only the pTol2{rps9::egfp}^frkt868^ donor construct with larvae co-injected with *tol2* mRNA, the co-injected larvae showed a stronger and less mosaic expression of EGFP, whereas plasmid-injected larvae only showed mosaic fluorescence, in addition to autofluorescence signal around the adult eyes (**Figure 3A, B**). Moreover, whereas individuals only injected with plasmid typically lost EGFP signal by three days of development, several co-injected zygotes gave rise to fully fluorescent adults (**Figure 3C, D**). These findings establish that co-injection of *tol2* mRNA enhances the stable inheritance of constructs throughout cell divisions.

**Figure 4:**
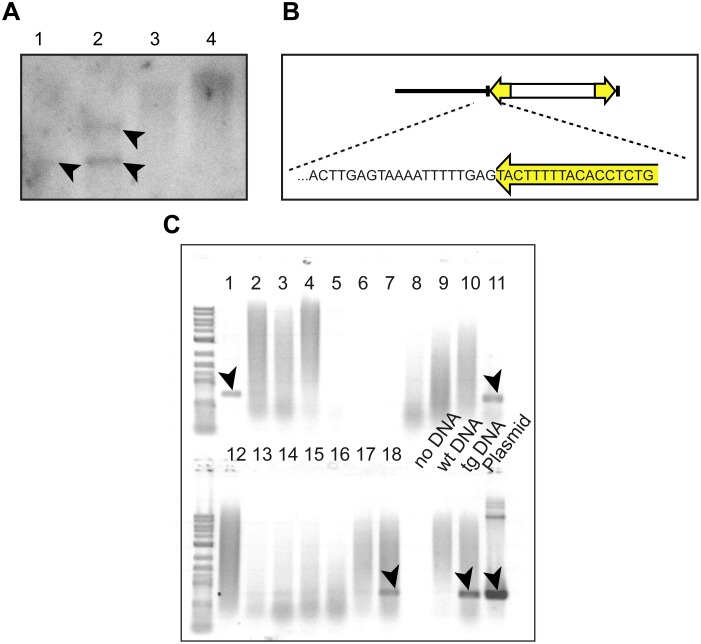
Genomic integration and inheritance of Tol2-mediated constructs. (**A**) Genomic Southern blot with an *egfp* probe reveals genomic integrations of pTol2{rps9::egfp}^frkt868^ in tested G_0_ individuals (arrows). (**B**) Precise genomic integration of a the reporter construct at the end of the IR as revealed by sequencing of an amplicon including neighboring genomic DNA, generated using Tol2IR-specific TAIL-PCR primers. (**C**) Integration and inheritance of *egfp* in individual G_0_ and G_1_ animals: PCR amplicons specific for *egfp* (arrowheads) can be detected in genomic DNA of both G_0_ (lanes 9–18) and G_1_ (lanes 1–8) individuals. Controls: no DNA (lane 19), wild-type gDNA (lane 20), gDNA extracted from a tuba::egfp^vbci1^ transgenic individual (lane 21), reporter construct plasmid DNA (lane 22).

In other species such as the nematode *Caenorhabditis elegans* or the mouse, exogenous DNA can be inherited in an extrachromosomal fashion [33], [34]. Therefore, we assessed next if injected individuals integrated copies of the reporter construct in their nuclear genomes. A radiolabeled *egfp* probe detected specific bands in a Southern hybridization with genomic DNA isolated from injected individuals (**Figure 4A**). Moreover, we could amplify by TAIL-PCR non-vector sequence flanking one of the Tol2 repeats from the same samples (**Figure 4B**). The non-vector sequence was precisely juxtaposed next to the Tol2 repeat where integration into the genome would be expected to occur. Together, these findings establish that Tol2 mediates excision and integration of reporter constructs into the *Platynereis* genome. The recovery of fully fluorescent adults shows that integration of reporter constructs can occur early enough during development to generate transgenic animals with little or no mosaicism in the injected (G_0_) generation.

### Stable Expression after Germ-line Transmission of Mos1-, but not Tol2-mediated Insertions

Whereas Tol2-mediated transgenes showed robust expression in the injected generation of animals, we were unable to retrieve fluorescent offspring of Tol2-transposon-injected animals, even if they were pre-screened for fluorescence in larval stages (n = 168 tested parents). We hypothesize that this discrepancy between fluorescence in the two generations reflects a permanent silencing of the reporter, possibly by a mechanism residing in the germ line, which is known to be able to counteract transposons in other species [35], [36]. In line with this hypothesis, *egfp* coding sequence could still be amplified from 3 of 18 non-fluorescent batches derived from pre-screened parents (**Figure 4C**).

Co-injection of *mos1* RNA with pMos{rps9::egfp}^frkt1074^ only leads to a moderate frequency (5%) of green larvae when tested at 7 dpf, significantly lower than for Tol2 (18%, **Table 1**). Similar to Tol2, we observe genomic integration of Mos1-based constructs (see below, **Figure S1**) Together with the excision assay, these findings indicate that Mos1 cleaves co-injected DNA constructs, and that these constructs can integrate into the *Platynereis* genome, even though excision and/or transposition mechanisms seem to occur with reduced efficiency, and expression rates are consistently lower than the ones observed for Tol2-mediated transgenesis. Notwithstanding this lower efficiency, however, Mos1-mediated transgenes are both heritable and functional in subsequent generations (ref. [25] and see below).

**Table 1:** Efficiency of Tol2 and Mos1 transposon-mediated transient transgenesis.

	Tol2	Mos1
injected	1089	496
survivors	603	339
fluorescence	107 (18%)	16 (5%)

Efficiency is based on the detection of broad Egfp expression 7 days after injection of either pTol2{rps9::egfp}^frkt868^ (left column) or pMos{rps9::egfp}^frkt1074^ (right column). Percentages are calculated as the fraction of Egfp-expressing animals of total survivors for each of the constructs.

### Novel Reporters Targeting Distinct *Platynereis* Cell Types

**Figure 5:**
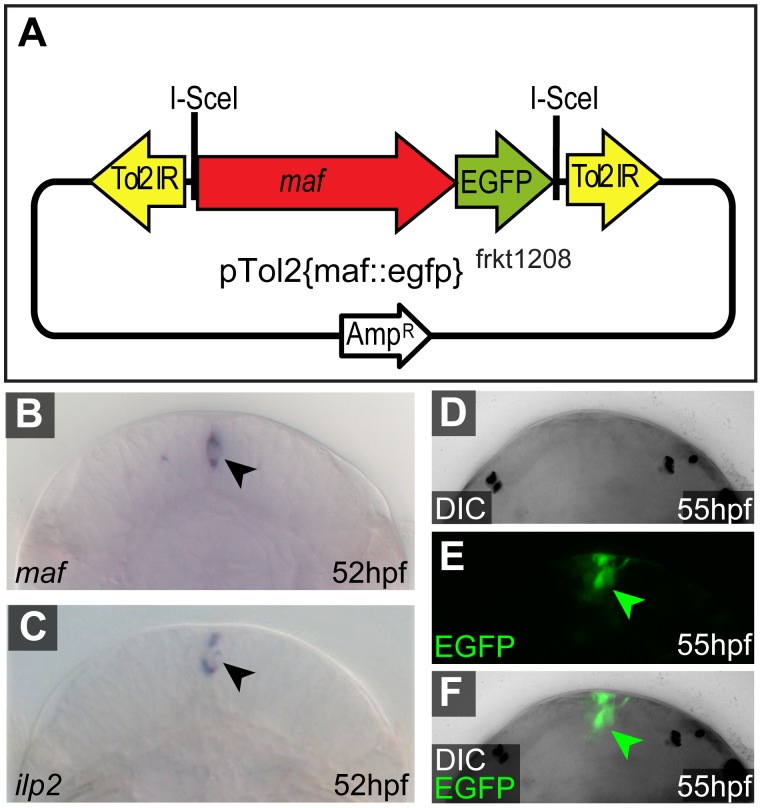
The *maf* locus drives EGFP in putative neurosecretory brain cells . (**A**) Scheme of donor vector pTol2{maf::egfp}^frkt1208^ containing a 3.6 kB upstream of the *maf* start codon (compare to Figure 1A). (**B**) Expression of endogenous *maf* RNA (arrowhead) in the medial central brain of early metatrochophore larvae. (**C**) Synexpression of the *insulin-like peptide 2*/*ilp2* mRNA in the same region (arrowhead), suggesting that *maf* demarcates neurosecretory cells. (D–F) EGFP expression driven by pTol2{maf::egfp}^frkt1208^ demarcates cells in the same region as endogenous *maf*.

**Figure 6:**
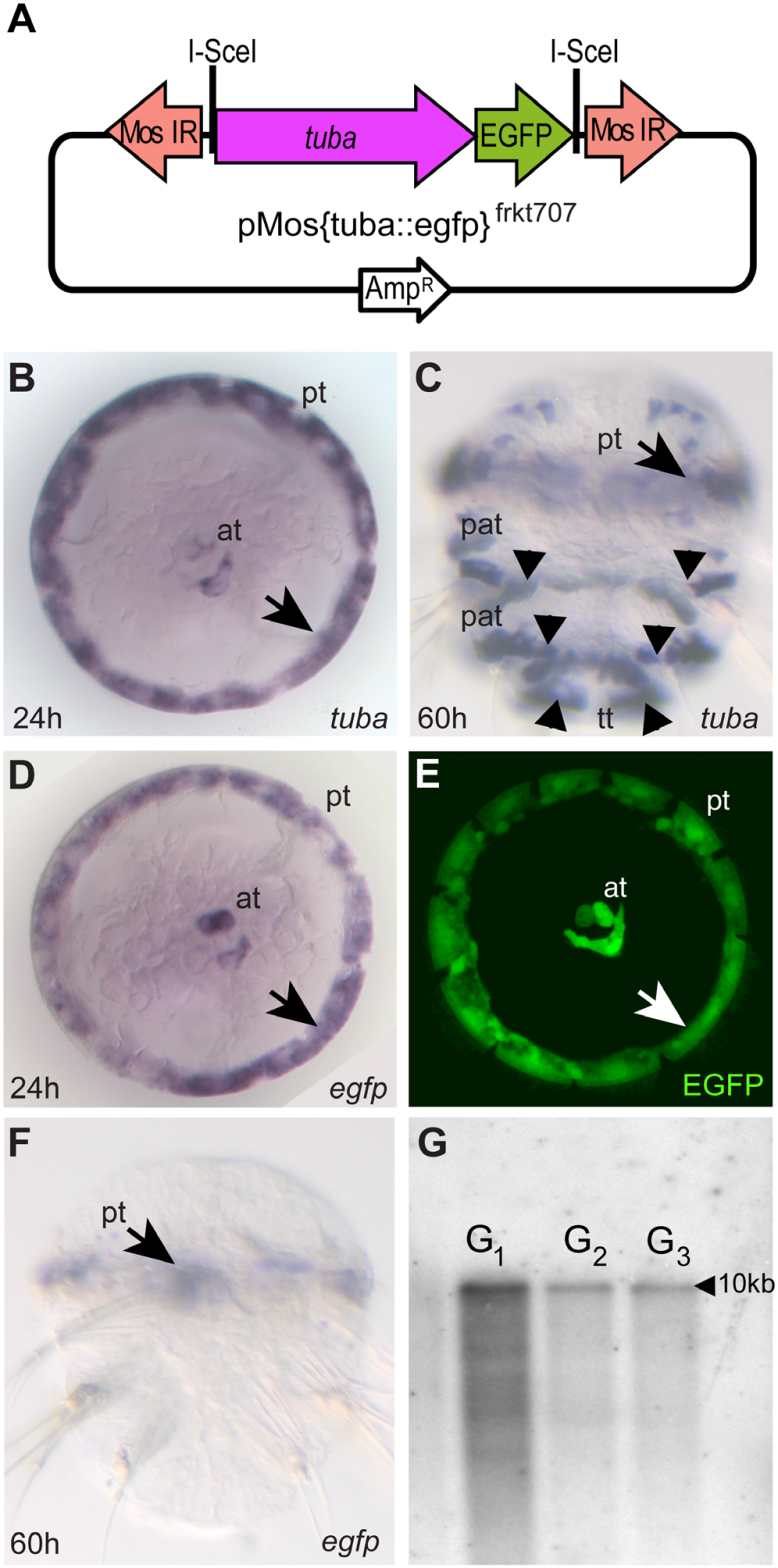
EGFP expression at the trochophore stage reflects endogenous *tuba* expression in tuba::egfp^vbci1^ animals. (**A**) Scheme of donor vector pMos{tuba::egfp}^frkt707^ containing 4.4 kB of DNA upstream of the *alpha-tubulin (tuba)* start codon (compare to Figure 2A). (**B, C**) Expression of endogenous *tuba* in the prototroch (pt; arrows), apical ciliary tuft (at) and paratrochs (pat; arrowheads) of *Platynereis* larvae at the trochophore (B) and metatrochophore (C) stages. (**D, E**) Expression of *egfp* RNA (D) and EGFP protein in tuba::egfp^vbci1^ trochophore larvae perfectly reflects endogenous *tuba* expression. (**F**) *egfp* expression in tuba::egfp^vbci1^ metatrochophore larvae remains restricted to the prototroch (arrow); (**G**) Stable inheritance of the construct, as evidenced by Southern blots of carriers from three different generations, probed with a labeled *egfp* fragment.

Whereas cis-regulatory regions of ubiquitously expressed genes are useful to globally misexpress genes, an important application of transgenic reporters for evolutionary and developmental studies is also to label specific cell types. In addition to the ubiquitous *rps9* gene (this study) and the specific *r-opsin* gene [25], [30], we therefore selected two genes expressed in distinct subpopulations of cells: a specific alpha-tubulin gene (*tuba*) characteristic for larval cells bearing motile cilia, and the gene *maf* that demarcates specific neurons in the larval brain that are distinct from the *r-opsin*-positive photoreceptors. To generate expression constructs for these genes, a 3.6 kB fragment upstream of the *maf* start codon was fused to the *egfp* coding sequence, and the resulting fragment cloned into the transposon target vectors. Likewise, 4.4 kB of the *tuba* locus upstream of the first coding exon were fused to the *egfp* coding sequence, and the resulting fragment cloned into the target vectors. For maf::egfp, we report here on the transient expression from the Tol2-based construct pTol2{maf::egfp}^frkt1208^ (**Figure 5A**). For tuba::egfp, we describe a stable strain that carries an integration of pMos{tuba::egfp}^frkt707^ (**Figure 6A**).

*Platynereis maf* encodes a transcription factor orthologous to *Drosophila* Traffic-jam and vertebrate c-Maf, Maf-A and Maf-B. When tested at 52 hpf, we detect 1–3 *maf-*expressing cells in a cluster in the medial larval brain, located below the apical tuft (**Figure 5B**). By position, these *maf*-positive cells likely co-express *ilp2*, a *Platynereis* insulin-like peptide precursor (**Figure 5C**). This constellation is reminiscent of the role of vertebrate large Maf members in the regulation of insulin in pancreatic beta cells [37], and suggests that the *maf-*positive *Platynereis* brain cells are neurosecretory.

Consistent with the endogenous expression of *maf*, embryos co-injected with pTol2{maf::egfp}^frk1208^ plasmid and *tol2* mRNA displayed EGFP in cells of the central larval brain. Detailed analyses of EGFP fluorescence by confocal microscopy (performed at around 55 hpf) in three independent specimens consistently revealed a cluster of cells in the central medial brain that by position correlates to the medial cells observed in the *maf* in situ hybridization (**Figure 5D–F**). These analyses also reveal projections of the EGFP-labeled cells. The observation of these projections is consistent with the notion that the reporter demarcates neurosecretory cells. Together with our observations for the r-opsin::egfp strain [25], these data attest to the ability of cell-specific reporters to highlight cellular processes in *Platynereis*.

For the analysis of tuba::egfp, we raised zygotes co-injected with pMos{tuba::egfp}^frkt707^ and *mos1* RNA to adults, and investigated offspring of these animals for fluorescent expression. 2 of 23 analyzed adults yielded fluorescent batches. One of these could be propagated further. Together with the respective figures for the r-opsin::egfp strain, this leads to an average rate of fluorescent offspring of 5% (**Table 2**), compatible with the rate of Mos1-mediated transgenesis observed in our transient expression assays (**Table 1**). We refer to the analyzed tuba::egfp strain as tuba::egfp^vbci1^.

**Table 2:** Survival and transmission rates for the injection of Mos1-based fluorescent reporter constructs.

reporter construct	injected	mature G_0_	transgenic G_1_
tuba::egfp	409	23	2
r-opsin::egfp	363	39	1
total	772	62 (8% of injected)	3 (5% of mature)

Data are shown for both pMos{tuba::egfp}^frkt707^ and pMos{r-opsin::egfp}^frkt890^ injections. Injected zygotes (left column) were raised to mature animals (G_0_; middle column), and EGFP expression in the offspring (G_1_; right column) was monitored. Survival rate and transgenic founder rate are given as percentage of injected animals and mature animals, respectively.

In trochophore larvae, endogenous *tuba* is prominently expressed in the prototroch cells, and also shows a specific expression in the cells of the apical ciliary tuft (**Figure 6B**). Subsequently, *tuba* is also expressed in the emerging posterior ciliary bands, referred to as paratrochs (**Figure 6C**). In tuba::egfp^vbci1^ trochophore larvae, *egfp* mRNA expression as well as protein fluorescence precisely matches the *tuba* expression in prototroch and apical ciliary tuft (**Figure 6D, E**). EGFP protein also labels the ciliary extensions of the prototroch, underlining the suitability of transgenic strains for the analysis of subcellular structures. In contrast to endogenous *tuba* mRNA, *egfp* mRNA is not observed in the paratroch cells, and expression in the prototroch is diminished over the course of metatrochophore development (**Figure 6F**). As the expression of *maf::egfp* as well as *r-opsin::egfp* [25] attest to, fluorescence is not generally restricted to early stages of *Platynereis* development. We therefore hypothesize that the *tuba::egfp* reporter only contains a subset of the regulatory elements required to maintain the full expression pattern, or that it is integrated in the vicinity of a repressor element impeding the enhancer’s late larval expression.

Finally, to investigate the stability of the integrated construct, we outbred G_1_ animals for additional two generations, selecting for carriers based on their EGFP expression at 24 hpf. Trochophores in the G_3_ generation still showed expression in the same pattern. Likewise, Southern analysis on digested genomic DNA from G_1_, G_2_, and G_3_ carriers of the tuba::egfp^vbci1^ strain revealed a single *egfp*-positive bands of the unchanged size in each specimen (**Figure 6G**). Together, this argues that construct DNA is inserted in a single locus, inherited in a stable fashion, and does not re-mobilize on its own from the integration locus. To investigate the nature of the integration further, we used TAIL-PCR with construct-specific primers to isolate neighboring DNA. Consistent with the Southern analysis, we were able to isolate at least one stretch of genomic DNA from the tuba::egfp^vbci1^ strain (**Figure S1A, Alignments S3** and **S4**), and confirmed the presence of this genomic DNA next to the reporter by specific PCR. The junction between reporter and genome, however, was offset by 40 bp from the proper 3′ITR (**Alignment S4**), consistent with the notion that the inserted fragment was target of exonuclease activity, similarly to the conclusions for the excision assay. It therefore also seems unlikely that this arm of the construct was inserted by a canonical transposition mechanism, even though the detected genomic sequence is compatible with the characteristic TA signature found in Mos1 insertions (**Alignment S4**). In further support of a non-canonical insertion mechanism, we find evidence for multiple insertions in both tested strains: From the same tuba::egfp^vbci1^ individuals that yielded the reporter-to-genome junction, additional amplicons can be recovered that contain the 3′ITR and flanking vector sequence instead of genomic DNA, thus revealing the presence of at least one additional copy of the 3′ arm of the construct. Given the results of the Southern analysis, the most plausible explanation is that there are at least two reporter fragments that are integrated in close proximity to each other. Similarly, one of the fragments recovered from the 3′ arm of the rops::egfp^vbci2^ strain reveals a juxtaposition of 3′ vector sequence with *dsRed* coding sequence. As *dsRed* is included as an internal marker gene in the 5′ end of the reporter, these data support the notion that two reporter fragments are juxtaposed in the genome (**Figure S1B**).

Together, our data reveal an unexpected impairment of the Tc1/mariner type Mos1 transposon system, both on the level of excision and integration. The reason of this impairment is still unclear. Despite its low efficiency, however, Mos1-mediated transgenesis appears to be a suitable tool for generating stable transgenic strains in *Platynereis*, whereas Tol2 represents the most efficient tool for transient transgenic applications.

## Discussion

Over the past years, *Platynereis* has become an important reference species for evolution and development. Our study extends the functional toolkit available for *Platynereis*, by establishing a second transposon system, Tol2, that yields more than threefold higher rates of transgenesis than the Mos1 system used to generate the first transgenic *Platynereis* strain [25]. Moreover, we demonstrate that Tol2-mediated transgenesis yields precise excision products and integrates in a canonical fashion, whereas co-injection of *mos1* mRNA with pMos donor constructs generates a panel of excision products that appear inconsistent with a standard cut-and-paste mechanism. The reasons for this discrepancy are currently unclear.

One contributing factor could be the difference in the excision mechanisms of Tol2 and Mos1: hAT transposases like Tol2 generate donor products whose 5′ and 3′ ends are chemically linked as hairpins, whereas Mos1 generates double-strand breaks with open 5′ and 3′ ends [38]. Possibly, the chemical protection of the hairpin intermediates results in more faithful repair than the open double strand breaks produced by Mos1. Consistent with this notion, Mos1-triggered non-homologous end-joining can lead to insertions and deletions in *Caenorhabditis elegans* [39]; likewise, our recent work indicates that in *Platynereis* genomic DNA, open double-strand breaks generated by engineered TALE nucleases can lead to insertions and deletions, presumably also by non-homologous end-joining repair [40].

Such repair-induced artifacts might explain the observed insertions, and part of the observed deletions, in the donor amplicons recovered from injected animals. However, non-homologous end joining repair alone cannot explain the recovery of donor amplicons in which parts of the Mos1 ITRs are still included. We speculate that these events may rather reflect some abandoned transposon intermediates, possibly after nicking at individual ITRs without complete paired-end complex formation. The occurrence of such intermediates has been proposed to occur in Mos1 transposition [41], even though the order of cleavage and complex formation remains debated (see e.g. ref. [42]). Adding to this, it is known that Mos1 as well as other Tc1 transposons are sensitive to large cargo sizes (see ref. [43] and references therein). The current design of the pMos donor [25] still contains around 1.5 kbp of sequence relevant for expression of a dsRed marker [44] that is not used in the *Platynereis* system. The resulting larger insert sizes may therefore favor such abandoned reactions over paired end complex formation and canonical excision.

Because the ubiquitous Tol2 construct we studied failed to express after germline transmission, we currently regard the two transposon systems as complementary approaches for *Platynereis* transgenesis, with Mos1 being the preferred system for stable transgenesis. Given the evidence that Mos1 only possesses low efficiency in *Platynereis*, decrease of cargo sizes, and additional changes that are known to enhance Mos1 efficiency, such as optimization of the inverted terminal repeats [45], could be useful to implement. Concerning the apparent silencing of the Tol2 construct, we have not yet addressed if its severity depends on specific properties of the pTol2{rps9::egfp} vector, such as its strong and ubiquitous expression, or on the proximity of the Tol2 inverted terminal repeats, and would therefore be relieved when using larger inserts. Of note, Tol2 is less sensitive to cargo sizes than Tc1 elements [43]. Future re-engineering might therefore be able to combine the advantages of both systems.

Our results have three additional implications. First, transient transgenic technology as pioneered in this study is a critical step towards the systematic assessment of the cis-regulatory activity of DNA fragments. Cis-regulatory assays are key for dissecting gene-regulatory networks [46], and are well established in other models in which experimental gene regulatory analyses are routinely being performed (see e.g. refs. [47], [48] and references therein). As an annelid representative, *Platynereis* is phyletically well positioned to allow cross-comparisons with other animals. Moreover, previous results argue that *Platynereis* has conserved ancient-type genome features and cell types, leading to the hypothesis that regulatory analyses in this species might be particularly useful to reconstruct the evolution of gene-regulatory networks [9]. Our data indicate that Tol2-mediated transgenesis is more effective than Mos1-mediated transgenesis for transient expression studies. However, the transient transgenesis efficiency of around 18% that we observe is still below the ones reported for established systems in which transient gene-regulatory analyses are performed, such as zebrafish (30–50%; [49]), medaka (47%; [50]) or *Ciona* (20–36%; [51]). Therefore, additional optimization of the current protocol will be useful to improve the throughput that can be achieved by the method. For instance, if the availability of functional transposase protein in the early cleavage stages is limiting transgenesis efficiency, it could be advantageous to generate a stable transgenic strain expressing *tol2* in the *Platynereis* oocyte to enhance the frequency of integration events. Such a strain might also be useful for remobilization of Tol2-inserted elements, as a potential strategy for enhancer detection [52]. Moreover, preliminary experiments have shown that phiC31 integrase can efficiently mobilize reporter constructs from co-injected DNA, but a suitable strain with a landing site to probe insertion frequencies has not yet been established (Backfisch and Raible, unpublished).

Second, whereas molecular research into larval *Platynereis* development is well advanced and allows gene expression studies with cellular resolution [18], [53], both transient and stable transgenic animals provide unprecedented access also to postlarval stages. Both of the novel cell-specific reporters presented in our study (*tuba::egfp* and *maf::egfp*) followed a simple design that could be achieved by fusion PCR. The versatility of the engineered vectors, as well as the growing genome resources for *Platynereis* [26] provide a good basis for the generation of additional constructs targeting different cell types. For more complex loci, a high-coverage genomic BAC library [20] is available that can be used for the generation of reporters by a recombineering approach [25]. Combined with molecular fingerprinting [9] or specific ablation [30], [54] of labeled cells, stable transgenic strains will enable future research into the characterization of specific cell types, and will help to test their requirement for physiology or development.

Finally, the results presented here do not only improve research in *Platynereis* itself, but also have potential impact on research in other animals. On the one hand, due to the scarcity of molecularly accessible species among annelids and other lophotrophozoans, *Platynereis* may serve as a reference organism in which genes of related species could be expressed and analysed, similar to the role that a conventional molecular model like *Drosophila melanogaster* takes on for the larger group of dipterans. On the other hand, we anticipate that the protocols developed here will also guide other researchers to pioneer transgenesis in their respective models. In particular, the contrast in transposon system efficiency for transient vs. stable transgenesis that we report may serve as an important caveat for researchers interested in pioneering transgenesis in other emerging model species. Our data suggest that the most efficient systems for transient transgenesis do not necessarily yield the best results for stable transmission of functional reporter constructs, and that therefore various systems should be considered.

## Materials and Methods

### Animal Culture

Animals were raised and bred in the MFPL marine facility according to established procedures [55]. Animal research and transgenic work followed applicable legislation, as also approved by the Max F. Perutz Laboratories committee for biological safety (session from Feb 19, 2009).

### Generation of Reporter Constructs

In analogy to the pMos-based vector pMosSce^frkt318^ [25], the vector pTol2Sce^frkt761^ was generated by introducing two SceI sites in head-to-head orientation next to the XhoI and KpnI cloning sites of pDestTol2pA [56].

To generate the *rps9::egfp* reporter constructs pMos{rps9::egfp}^frkt1074^ and pTol2{rps9::egfp}^frkt868^, the sequence upstream of the *Platynereis rps9* coding sequence was determined by sequencing fragments generated by *rps9*-specific TAIL-PCR (see below) on genomic DNA. A 1.6 kbp fragment upstream of the start codon of *rps9* was amplified using primers rF and rL (for all primer sequences see **Table S1**). The resulting amplicon was linked by fusion PCR to the *egfp* coding sequence derived from pEGFP-N1 (Clontech) using primers gF and gL, and subcloned into the SceI sites of the pMosSce^frkt318^ and pTol2Sce^frkt761^ destination vectors.

To generate the *tuba::egfp* reporter construct pMos{tuba::egfp}^frkt707^, a 4.4 kb fragment of the *Platynereis tuba* gene upstream of the putative start codon was PCR-amplified from purified BAC-DNA (CH305_105D17) [20] using primers tF and tL. Fusion to *egfp* and further subcloning followed the same procedure as outlined for *rps9::egfp*.

To generate the maf::egfp reporter construct pTol2{maf::egfp}^frkt1208^, a 3.6 kB fragment upstream of the *maf* start codon was amplified by PCR using primers mF and mL, linked by fusion PCR to *egfp* coding sequence amplified using gF2 and gL, and subcloned into pTol2Sce^frkt761^ as outlined above.

### Microinjection of Zygotes

Freshly fertilized eggs were incubated for 45 minutes at 18°C. Zygotes were prepared for microinjection by washing off the jelly coat with sea water followed by Proteinase K treatment (Merck, 50 μg/ml final concentration) for 25 seconds to soften the egg envelope. After extensive washing with sea water, zygotes were transferred to an injection stage made of 1.5% agarose submerged in sea water. Microinjections were performed using a Leica LC2 microscope, a Transferman NK2 micromanipulator, a Femtojet express microinjector, and Femtotips II microcapillaries (all from Eppendorf). A detailed account of the microinjection protocol and the used stage will appear elsewhere (Tosches et al., in preparation). Zygotes were injected with a solution containing 0.2 μg/μl of endotoxin-free DNA, 0.2 μg/μl synthetic transposase mRNA, and 0.6% (w/v) TRITC-Dextrane (MW 70 k, Invitrogen). Injected zygotes were transferred into fresh sea water and incubated at 18°C for the indicated periods.

### Stainings and Microscopy

Whole-mount in-situ hybridizations of *Platynereis* larvae were performed after established protocols (ref. [21] and references therein). For improved staining, 10% (w/v) polyvinylalcohol was used in NBT-BCIP staining buffers. For imaging of live animals, *Platynereis* juvenile or adult worms were paralyzed by adding dropwise 1 M MgCl_2_ into sea water until no movement of the animals occurred.

A Zeiss Axioplan2 microscope with a 40x oil immersion objective and a Zeiss Axiocam MR5 camera was used for documentation of stainings from whole-mount in-situ-hybridisations. For live imaging, low magnification fluorescence images were taken with a Zeiss Lumar.V12 stereomicroscope with FITC fluorescence filter. Confocal images were taken using a Zeiss LSM 510 confocal microscope with a 488 nm excitation wavelength of an Argon laser. Images were processed using the ImageJ software package (http://imagej.nih.gov/ij/), to generate z-projections of scans.

### Transposase-activity Assay and TAIL-PCR

Primers T2f and T2r (**Table S1**) were designed to bind the pTol2Sce^frkt761^ backbone at 138/96 nt distance to the Tol2 inverted repeats. Primers M1f and M1r and nested primers M1nf and M1nr were designed to bind the pMosSce^frkt318^ backbone at 542/223 nt and 245/96 nt distance to the Mos1 repeats, respectively.

Experimental zygotes were injected as described, controls received only DNA and TRITC-Dextrane. Four hours after injection, three embryos per sample were collected in 1 μl of sea water and transferred into PCR mix containing 1xCl buffer (Qiagen), 0.4 nM/μl dNTP-mix, 1.25 U Hot star Taq DNA Polymerase (Qiagen), 0.25 pM/μl of each of the two primers corresponding to the injected vector. PCR for excision assays was performed under following conditions: 95°C - 5 min, 35× (95°C - 30 s, 60°C - 30 s, 72°C - 1 min), 72°C - 10 min. Amplicons were separated by gel electrophoresis, cut out, subcloned and sequenced.

Thermal asymmetric interlaced polymerase chain reaction (TAIL-PCR) was performed as described [11], [31]. The sequences of the specific primers are given in **Table S1**, with the following identities: RT1–RT3: primers for *rps9*; TL1-3, TR1-3: primers located close to the left and right terminal repeats of the Tol2 vector, respectively; ML1-3, MR1-3: primers located close to the left (3′ITR) and right (5′ITR) terminal repeats of the Mos1 construct, respectively. VL1-3: primers located in the donor vector outside the Mos1 3′ITR. Recovery of the genomic DNA displayed in **Figure S1B**/**Alignment S5** was independently validated by specific PCR with primers gDf and ML1.

### Southern Hybridization

Genomic DNA from samples was extracted using the Nucleospin tissue kit (Macherey-Nagel). 8 μg of genomic DNA per sample were digested at 37°C for 16 h using HindIII, analyzed on 0.8% agarose/1xTAE gel and subsequently blotted on nylon membrane (Peqlab). An *egfp* fragment was excised from pEGFP-N1 (Clontech) and radiolabeled with alpha-^32^P dCTP using the Radprime DNA labeling System (Invitrogen) for use as a probe in Southern blot hybridization. Blots were hybridized in Rapid Hyb buffer (GE Healthcare) at 65°C overnight. Membranes were washed once in 2xSSC/0.1%SDS for 20 min and twice in 0.2xSSC/0.1%SDS for 20 min before exposure on Phosphoimager screens.

## Supporting Information

Figure S1 Evidence for reporter fragmentation and genomic integration in GFP-expressing strains. Schematized alignments representing TAIL-PCR amplicons recovered from tuba::egfp^vbci1^ (A) and rops::egfp^vbci2^ (B) in comparison with the respective donor plasmids pMos{tuba::egfp} and pMos{rops::egfp}. Consistent with representation in **Alignments S3, S4,** and **S5**, red arrows demarcate position of 5′ and 3′ITRs; blue arrows show position of TAIL PCR primers; (i) and (ii) demarcate two alternative 3′ regions recovered from tuba::egfp^vbci1^ worms, indicative of multiple local integrations or partial duplications; yellow boxes indicate regions juxtaposed to the amplified vector sequence: genomic DNA (A) or *dsred* coding sequence (B) that is normally located in the 5′ region of the reporter sequence.
(TIF)

Table S1 **Sequences of primers used in this study.** The table shows the sequences for all primers used in this study, along with the primer names.
(DOCX)

Alignment S1 **Donor sequences recovered from** ***mos1*** **-injected worms (3′ arm).** Multiple sequence alignment showing sequences of fragments recovered from the excision assay in comparison to the pMos{rps9::egfp}^frkt1074^ donor reference sequence (bottom). Mos1 3′ Inverted Terminal Repeats (3′ITR) are indicated with red arrows; see **Figure 2D** for overview. The alignment does not show the following insertions that lie between the displayed regions of the amplicons and the regions in **Alignment S2** and cannot be aligned to the reference sequence: 13_14-1_M13: GTCCCTT (7 bp); 7_12.2-2_M13: TGGA (4 bp); 11_12.2-1_M13: AACCCGGAATGACCATGCGCATCC (24 bp); 8_20.1-1_M13: AA (2 bp); 9_6.5-4_M13: A (1 bp); 5_6.5-3_M13: GGCTCC (6 bp); 10_7.2-2_M13: TAATTAGACAAAGTGAAG (18 bp).
(PDF)

Alignment S2 **Donor sequences recovered from** ***mos1*** **-injected worms** **(5′ arm).** Multiple sequence alignment showing sequences of fragments recovered from the excision assay in comparison to the pMos{rps9::egfp}^frkt1074^ donor reference sequence (bottom). Mos1 5′ Inverted Terminal Repeats (5′ITR) are indicated with red arrows. See **Figure 2D** for overview.
(PDF)

Alignment S3 **TAIL-PCR amplicon recovered from stable tuba::egfp^vbci1^ animals (5′ arm).** Multiple sequence alignment showing the sequence of a fragment recovered by TAIL-PCR from the stable tuba::egfp^vbci1^ strain. The Mos1 5′ITR is indicated by a red arrow; blue arrows demarcate position of TAIL-PCR primers.
(PDF)

Alignment S4 **TAIL-PCR amplicons recovered from stable tuba::egfp^vbci1^ animals (3′ arm).** Multiple sequence alignment showing the sequences of three fragment recovered by TAIL-PCR from the stable tuba::egfp^vbci1^ strain. The Mos1 3′ITR is indicated by a red arrow; blue arrows demarcate position of TAIL-PCR primers as well as the gDf primer used to independently confirm the recovered integration; yellow box indicates *Platynereis* genomic DNA at transition between reporter and genomic DNA.
(PDF)

Alignment S5 **TAIL-PCR amplicons recovered from stable rops::egfp^vbci2^ animals (3′ arm).** Multiple sequence alignment showing the sequences of five fragments recovered by TAIL-PCR from the stable rops::egfp^vbci2^ strain. The Mos1 3′ITR is indicated by a red arrow; blue arrows demarcate position of TAIL-PCR primers (two sets were used); yellow box indicates *dsred* cDNA that it present at a different region of the donor plasmid, indicative of fragmentation of the donor prior to integration.
(PDF)

## References

[1] Jenner RA, Wills MA (2007) The choice of model organisms in evo-devo. Nature reviews Genetics 8: 311–319. 10.1038/nrg2062 17339879

[2] Bolker J (2012) Model organisms: There’s more to life than rats and flies. Nature 491: 31–33. 23128209 10.1038/491031a

[3] Philippe H, Brinkmann H, Copley RR, Moroz LL, Nakano H, et al (2011) Acoelomorph flatworms are deuterostomes related to *Xenoturbella* . Nature 470: 255–258. 21307940 10.1038/nature09676 PMC4025995

[4] Struck TH, Paul C, Hill N, Hartmann S, Hosel C, et al (2011) Phylogenomic analyses unravel annelid evolution. Nature 471: 95–98. 21368831 10.1038/nature09864

[5] Kocot KM, Cannon JT, Todt C, Citarella MR, Kohn AB, et al (2011) Phylogenomics reveals deep molluscan relationships. Nature 477: 452–456. 21892190 10.1038/nature10382 PMC4024475

[6] Simakov O, Marletaz F, Cho SJ, Edsinger-Gonzales E, Havlak P, et al (2013) Insights into bilaterian evolution from three spiralian genomes. Nature 493: 526–531. 23254933 10.1038/nature11696 PMC4085046

[7] Thisse C, Thisse B (2008) High-resolution in situ hybridization to whole-mount zebrafish embryos. Nature Protocols 3: 59–69. 18193022 10.1038/nprot.2007.514

[8] Chintapalli VR, Wang J, Dow JA (2007) Using FlyAtlas to identify better *Drosophila melanogaster* models of human disease. Nature Genetics 39: 715–720. 17534367 10.1038/ng2049

[9] Arendt D (2008) The evolution of cell types in animals: emerging principles from molecular studies. Nature Reviews Genetics 9: 868–882. 10.1038/nrg2416 18927580

[10] Ivics Z, Izsvák Z (2010) The expanding universe of transposon technologies for gene and cell engineering. Mobile DNA 1: 25. 21138556 10.1186/1759-8753-1-25 PMC3016246

[11] Sasakura Y, Awazu S, Chiba S, Satoh N (2003) Germ-line transgenesis of the Tc1/mariner superfamily transposon Minos in *Ciona intestinalis* . Proc Natl Acad Sci USA 100: 7726–7730. 12788975 10.1073/pnas.1230736100 PMC164655

[12] Hozumi A, Mita K, Miskey C, Mates L, Izsvak Z, et al (2013) Germline transgenesis of the chordate Ciona intestinalis with hyperactive variants of sleeping beauty transposable element. Developmental Dynamics 242: 30–43. 23073965 10.1002/dvdy.23891

[13] Halanych K (2004) The new view of animal phylogeny. Annual Review of Ecology Evolution and Systematics 35: 229–256.

[14] Aguinaldo AM, Turbeville JM, Linford LS, Rivera MC, Garey JR, et al (1997) Evidence for a clade of nematodes, arthropods and other moulting animals. Nature 387: 489–493. 9168109 10.1038/387489a0

[15] The Schistosoma japonicum Genome Sequencing, Functional Analysis Consortium (2009) The *Schistosoma japonicum* genome reveals features of host-parasite interplay. Nature 460: 345–351. 19606140 10.1038/nature08140 PMC3747554

[16] Takeuchi T, Kawashima T, Koyanagi R, Gyoja F, Tanaka M, et al (2012) Draft genome of the pearl oyster *Pinctada fucata*: a platform for understanding bivalve biology. DNA research 19: 117–130. 22315334 10.1093/dnares/dss005 PMC3325083

[17] Tessmar-Raible K, Raible F, Christodoulou F, Guy K, Rembold M, et al (2007) Conserved sensory-neurosecretory cell types in annelid and fish forebrain: insights into hypothalamus evolution. Cell 129: 1389–1400. 17604726 10.1016/j.cell.2007.04.041

[18] Tomer R, Denes AS, Tessmar-Raible K, Arendt D (2010) Profiling by image registration reveals common origin of annelid mushroom bodies and vertebrate pallium. Cell 142: 800–809. 20813265 10.1016/j.cell.2010.07.043

[19] Kerner P, Simionato E, Le Gouar M, Vervoort M (2009) Orthologs of key vertebrate neural genes are expressed during neurogenesis in the annelid *Platynereis dumerilii* . Evolution and development 11: 513–524. 19754708 10.1111/j.1525-142X.2009.00359.x

[20] Raible F, Tessmar-Raible K, Osoegawa K, Wincker P, Jubin C, et al (2005) Vertebrate-type intron-rich genes in the marine annelid *Platynereis dumerilii* . Science 310: 1325–1326. 16311335 10.1126/science.1119089

[21] Tessmar-Raible K, Steinmetz PR, Snyman H, Hassel M, Arendt D (2005) Fluorescent two-color whole mount in situ hybridization in *Platynereis dumerilii* (Polychaeta, Annelida), an emerging marine molecular model for evolution and development. Biotechniques 39: 460–464. 16235555 10.2144/000112023

[22] Christodoulou F, Raible F, Tomer R, Simakov O, Trachana K, et al (2010) Ancient animal microRNAs and the evolution of tissue identity. Nature 463: 1084–1088. 20118916 10.1038/nature08744 PMC2981144

[23] González-Estávez C, Momose T, Gehring WJ, Saló E (2003) Transgenic planarian lines obtained by electroporation using transposon-derived vectors and an eye-specific GFP marker Proc Natl Acad Sci USA. 100: 14046–14051. 10.1073/pnas.2335980100 PMC283543 14615580

[24] Kines KJ, Morales ME, Mann VH, Gobert GN, Brindley PJ (2008) Integration of reporter transgenes into Schistosoma mansoni chromosomes mediated by pseudotyped murine leukemia virus. FASEB Journal 22: 2936–2948. 18403630 10.1096/fj.08-108308 PMC2493450

[25] Backfisch B, Veedin Rajan VB, Fischer RM, Lohs C, Arboleda E, et al (2013) Stable transgenesis in the marine annelid *Platynereis dumerilii* sheds new light on photoreceptor evolution. Proc Natl Acad Sci USA 110: 193–198. 23284166 10.1073/pnas.1209657109 PMC3538230

[26] Simakov O, Larsson TA, Arendt D (2013) Linking micro- and macro-evolution at the cell type level: a view from the lophotrochozoan *Platynereis dumerilii* . Briefings in functional genomics Sep 12(5): 430–439. 10.1093/bfgp/els049 23172798

[27] Grabher C, Wittbrodt J (2007) Meganuclease and transposon mediated transgenesis in medaka. Genome Biology 8 Suppl 1S10. 18047687 10.1186/gb-2007-8-s1-s10 PMC2106848

[28] Renfer E, Amon-Hassenzahl A, Steinmetz PR, Technau U (2010) A muscle-specific transgenic reporter line of the sea anemone, *Nematostella vectensis* . Proc Natl Acad Sci USA 107: 104–108. 20018670 10.1073/pnas.0909148107 PMC2806778

[29] Hobert O (2002) PCR fusion-based approach to create reporter gene constructs for expression analysis in transgenic *C. elegans* . Biotechniques 32: 728–730. 11962590 10.2144/02324bm01

[30] Veedin-Rajan VB, Fischer RM, Raible F, Tessmar-Raible K (2013) Conditional and Specific Cell Ablation in the Marine Annelid *Platynereis dumerilii* . PLoS One 8: e75811. 24086637 10.1371/journal.pone.0075811 PMC3782428

[31] Liu YG, Mitsukawa N, Oosumi T, Whittier RF (1995) Efficient isolation and mapping of Arabidopsis thaliana T-DNA insert junctions by thermal asymmetric interlaced PCR. The Plant journal 8: 457–463. 7550382 10.1046/j.1365-313x.1995.08030457.x

[32] Pavlopoulos A, Averof M (2005) Establishing genetic transformation for comparative developmental studies in the crustacean *Parhyale hawaiensis* . Proc Natl Acad Sci USA 102: 7888–7893. 15911760 10.1073/pnas.0501101102 PMC1142369

[33] Stinchcomb DT, Shaw JE, Carr SH, Hirsh D (1985) Extrachromosomal DNA transformation of *Caenorhabditis elegans* . Molecular and cellular biology 5: 3484–3496. 3837845 10.1128/mcb.5.12.3484-3496.1985 PMC369179

[34] Rassoulzadegan M, Leopold P, Vailly J, Cuzin F (1986) Germ line transmission of autonomous genetic elements in transgenic mouse strains. Cell 46: 513–519. 3015417 10.1016/0092-8674(86)90876-7

[35] Sijen T, Plasterk RH (2003) Transposon silencing in the *Caenorhabditis elegans* germ line by natural RNAi. Nature 426: 310–314. 14628056 10.1038/nature02107

[36] Ghildiyal M, Zamore PD (2009) Small silencing RNAs: an expanding universe. Nature Reviews Genetics 10: 94–108. 10.1038/nrg2504 PMC2724769 19148191

[37] Matsuoka TA, Zhao L, Artner I, Jarrett HW, Friedman D, et al (2003) Members of the large Maf transcription family regulate insulin gene transcription in islet beta cells. Molecular and cellular biology 23: 6049–6062. 12917329 10.1128/MCB.23.17.6049-6062.2003 PMC180917

[38] Zhou L, Mitra R, Atkinson PW, Hickman AB, Dyda F, et al (2004) Transposition of hAT elements links transposable elements and V(D)J recombination. Nature 432: 995–1001. 15616554 10.1038/nature03157

[39] Robert V, Bessereau JL (2007) Targeted engineering of the *Caenorhabditis elegans* genome following Mos1-triggered chromosomal breaks. The EMBO journal 26: 170–183. 17159906 10.1038/sj.emboj.7601463 PMC1782371

[40] Bannister S, Antonova O, Polo A, Lohs C, Hallay N, et al. (2014) TALENs mediate efficient and heritable mutation of endogenous genes in the marine annelid *Platynereis dumerilii*. Genetics. Early online March 20; doi:10.1534/genetics.113.161091. 10.1534/genetics.113.161091 PMC4012502 24653002

[41] Dawson A, Finnegan DJ (2003) Excision of the *Drosophila* mariner transposon Mos1. Comparison with bacterial transposition and V(D)J recombination. Molecular Cell 11: 225–235. 12535535 10.1016/s1097-2765(02)00798-0

[42] Cuypers MG, Trubitsyna M, Callow P, Forsyth VT, Richardson JM (2013) Solution conformations of early intermediates in Mos1 transposition. Nucleic acids research 41: 2020–2033. 23262225 10.1093/nar/gks1295 PMC3561948

[43] Mátés L, Izsvák Z, Ivics Z (2007) Technology transfer from worms and flies to vertebrates: transposition-based genome manipulations and their future perspectives. Genome Biology 8 Suppl 1S1. 18047686 10.1186/gb-2007-8-s1-s1 PMC2106849

[44] Horn C, Wimmer EA (2000) A versatile vector set for animal transgenesis. Development genes and evolution 210: 630–637. 11151300 10.1007/s004270000110

[45] Jaillet J, Genty M, Cambefort J, Rouault JD, Augé-Gouillou C (2012) Regulation of mariner transposition: the peculiar case of Mos1. PloS One 7: e43365. 22905263 10.1371/journal.pone.0043365 PMC3419177

[46] Davidson EH (2006) The regulatory genome: Gene regulatory networks in development and evolution. San Diego: Academic Press.

[47] Allende ML, Manzanares M, Tena JJ, Feijóo CG, Gómez-Skarmeta JL (2006) Cracking the genome’s second code: enhancer detection by combined phylogenetic footprinting and transgenic fish and frog embryos. Methods 39: 212–219. 16806968 10.1016/j.ymeth.2005.12.005

[48] Nam J, Dong P, Tarpine R, Istrail S, Davidson EH (2010) Functional cis-regulatory genomics for systems biology. Proc Natl Acad Sci USA 107: 3930–3935. 20142491 10.1073/pnas.1000147107 PMC2840491

[49] Bessa J, Tena JJ, de la Calle-Mustienes E, Fernandez-Miñán A, Naranjo S, et al (2009) Zebrafish enhancer detection (ZED) vector: a new tool to facilitate transgenesis and the functional analysis of cis-regulatory regions in zebrafish. Developmental Dynamics 238: 2409–2417. 19653328 10.1002/dvdy.22051

[50] Mongin E, Auer TO, Bourrat F, Gruhl F, Dewar K, et al (2011) Combining computational prediction of cis-regulatory elements with a new enhancer assay to efficiently label neuronal structures in the medaka fish. PLoS One 6: e19747. 21637758 10.1371/journal.pone.0019747 PMC3103512

[51] Sasakura Y, Oogai Y, Matsuoka T, Satoh N, Awazu S (2007) Transposon mediated transgenesis in a marine invertebrate chordate: *Ciona intestinalis* . Genome biology 8 Suppl 1S3. 18047695 10.1186/gb-2007-8-s1-s3 PMC2106840

[52] Sasakura Y, Konno A, Mizuno K, Satoh N, Inaba K (2008) Enhancer detection in the ascidian *Ciona intestinalis* with transposase-expressing lines of Minos. Developmental Dynamics 237: 39–50. 17948255 10.1002/dvdy.21333

[53] Asadulina A, Panzera A, Verasztó C, Liebig C, Jékely G (2012) Whole-body gene expression pattern registration in Platynereis larvae. EvoDevo 3: 27. 23199348 10.1186/2041-9139-3-27 PMC3586958

[54] Jekely G, Colombelli J, Hausen H, Guy K, Stelzer E, et al (2008) Mechanism of phototaxis in marine zooplankton. Nature 456: 395–399. 19020621 10.1038/nature07590

[55] Hauenschild C, Fischer A (1969) *Platynereis dumerilii* Mikroskopische Anatomie, Fortpflanzung und Entwicklung [*Platynereis dumerilii*. Microscopical anatomy, reproduction and development]. Grosses Zoologisches Praktikum. Stuttgart: G. Fischer Verlag.

[56] Kwan KM, Fujimoto E, Grabher C, Mangum BD, Hardy ME, et al (2007) The Tol2kit: a multisite gateway-based construction kit for Tol2 transposon transgenesis constructs. Developmental Dynamics 236: 3088–3099. 17937395 10.1002/dvdy.21343

