# Supplementary figures and images for: Tools for Gene-Regulatory Analyses in the Marine Annelid Platynereis dumerilii
Source: PLoS One. 2014 Apr 8;9(4):e93076. doi: 10.1371/journal.pone.0093076 (PMC3979674; doi:10.1371/journal.pone.0093076)

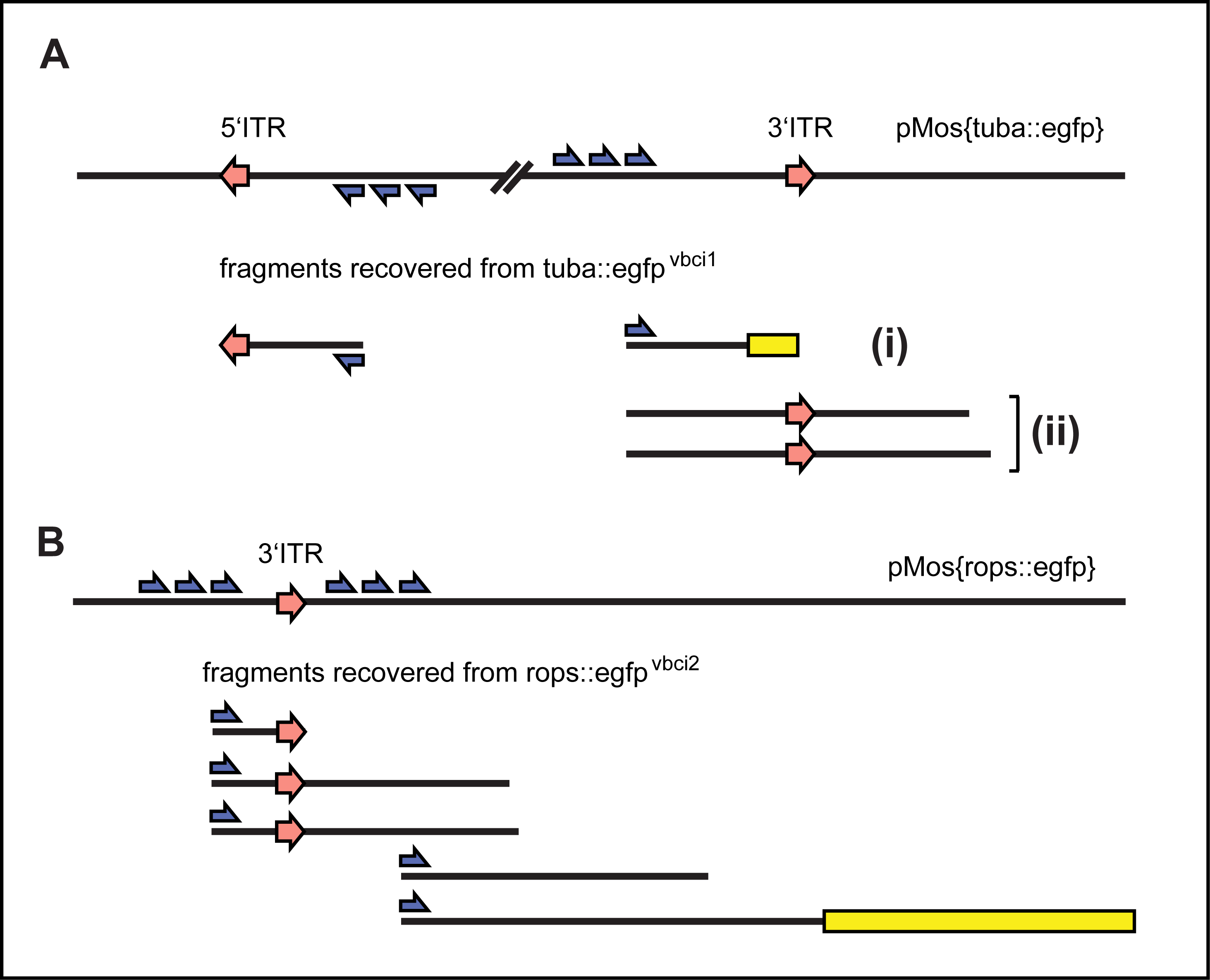

Supplement: Figure S1 — Evidence for reporter fragmentation and genomic integration in GFP-expressing strains. Schematized alignments representing TAIL-PCR amplicons recovered from tuba::egfpvbci1 (A) and rops::egfpvbci2 (B) in comparison with the respective donor plasmids pMos{tuba::egfp} and pMos{rops::egfp}. Consistent with representation in Alignments S3, S4, and S5, red arrows demarcate position of 5′ and 3′ITRs; blue arrows show position of TAIL PCR primers; (i) and (ii) demarcate two alternative 3′ regions recovered from tuba::egfpvbci1 worms, indicative of multiple local integrations or partial duplications; yellow boxes indicate regions juxtaposed to the amplified vector sequence: genomic DNA (A) or dsred coding sequence (B) that is normally located in the 5′ region of the reporter sequence. (TIF) [file pone.0093076.s001.tif]

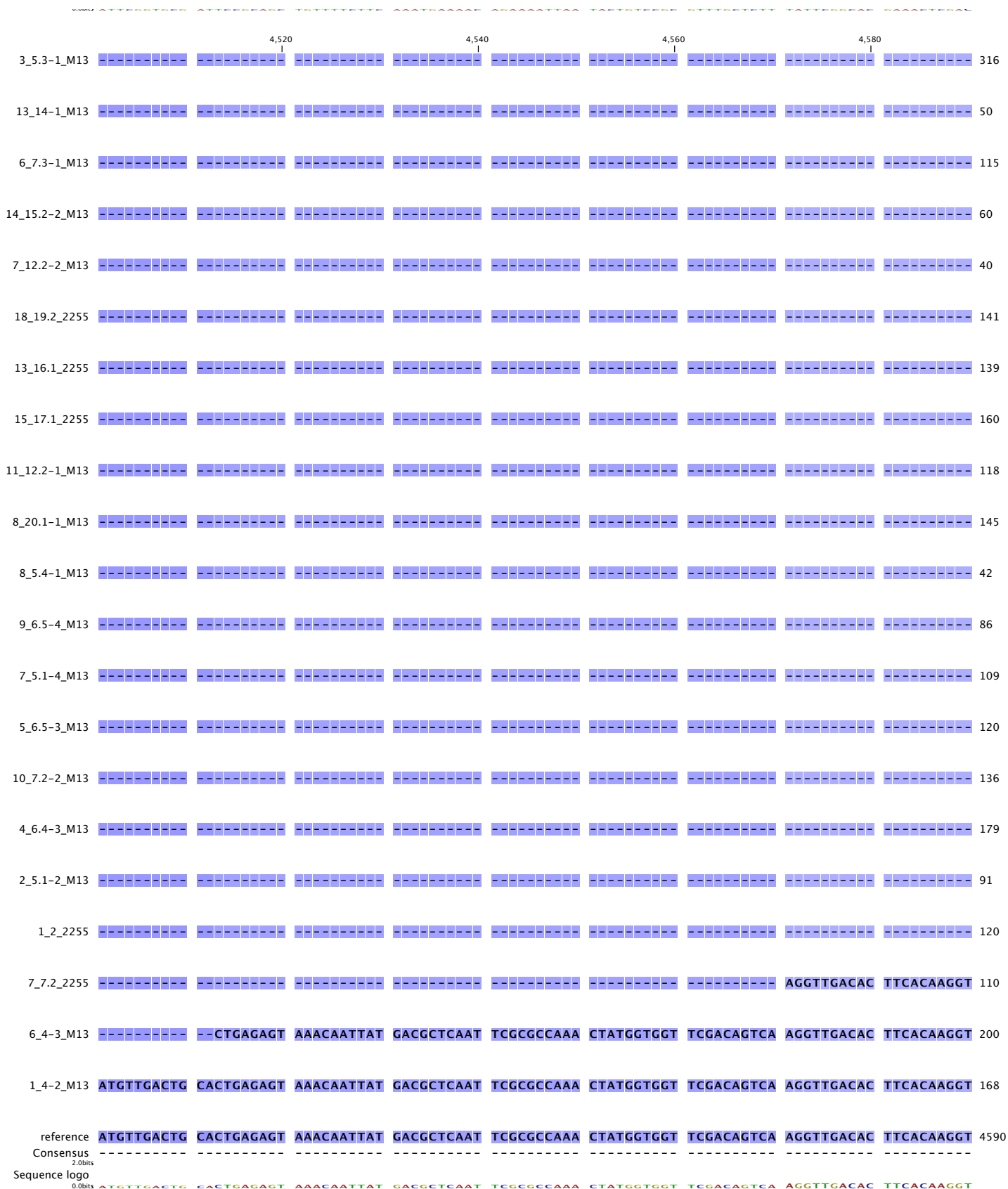

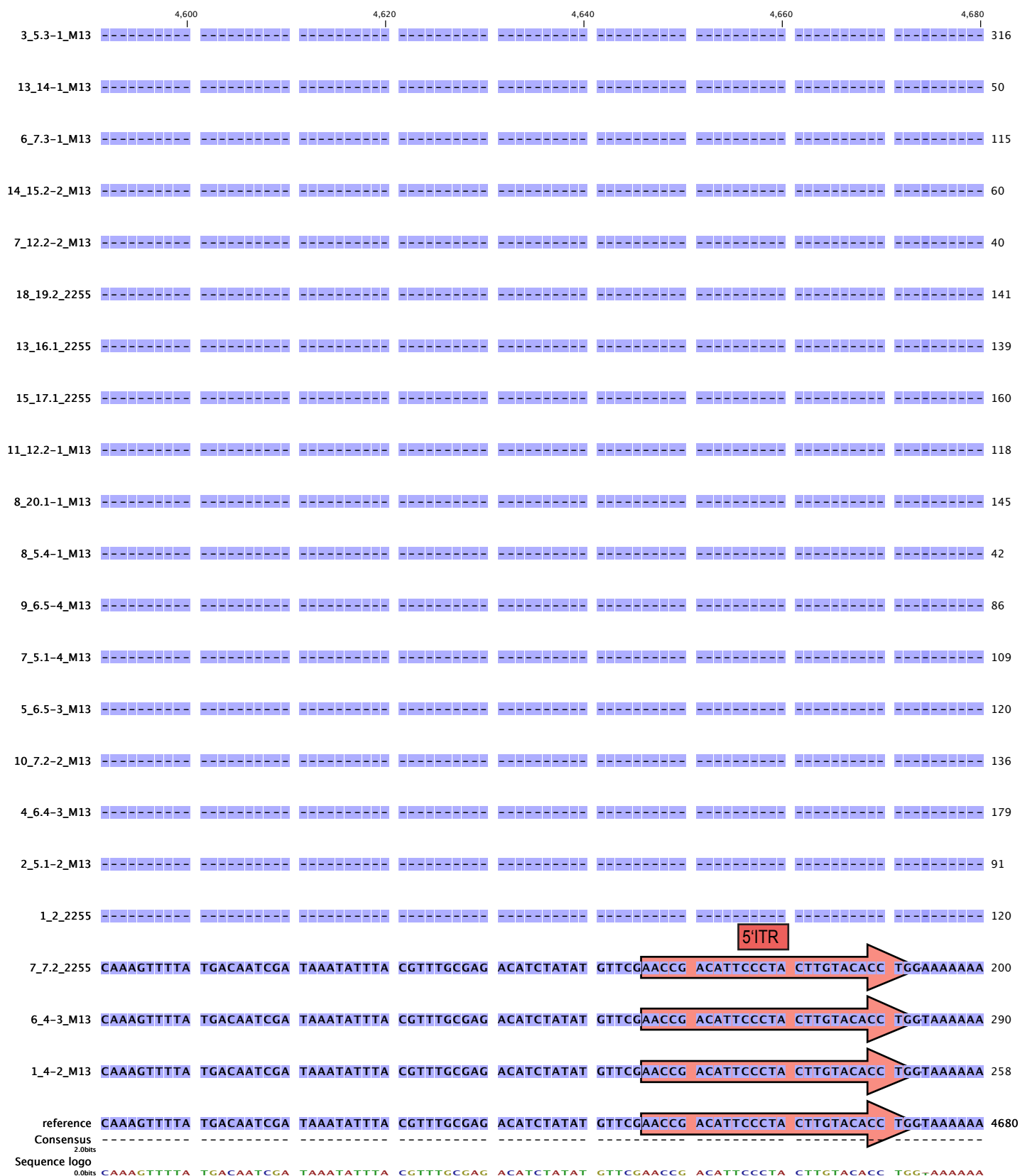

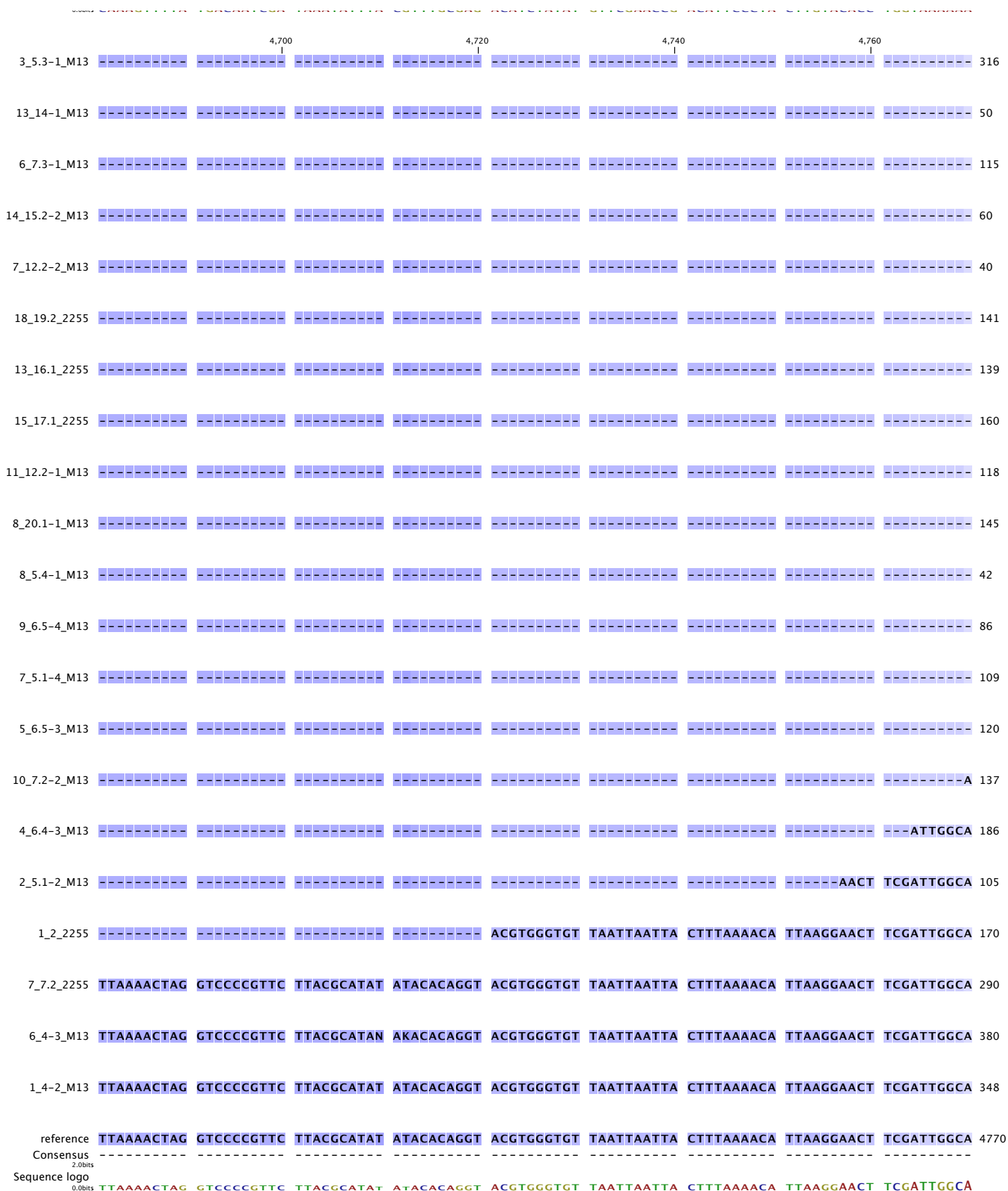

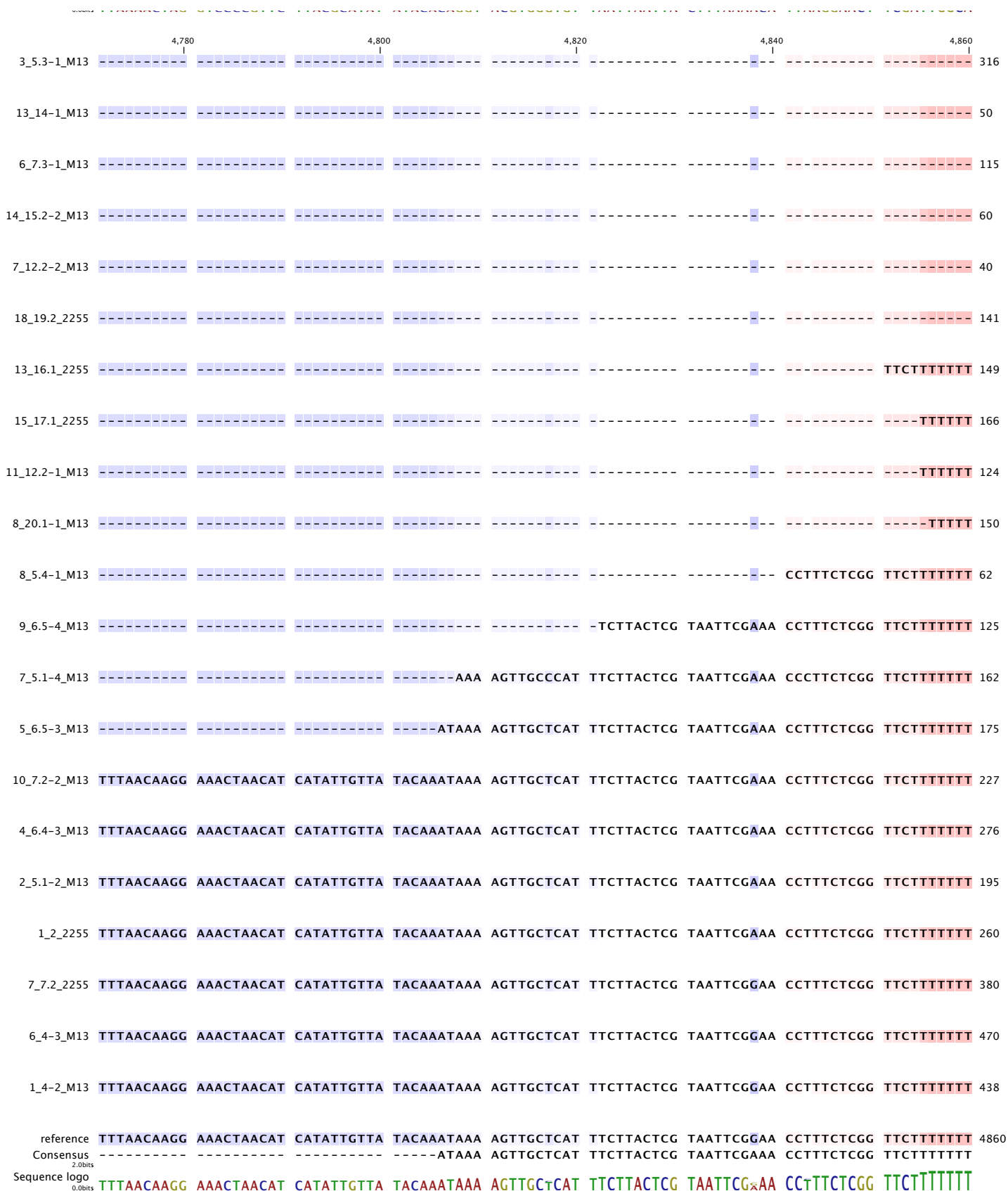

Supplement: Alignment S2 — Donor sequences recovered from mos1 -injected worms (5′ arm). Multiple sequence alignment showing sequences of fragments recovered from the excision assay in comparison to the pMos{rps9::egfp}frkt1074 donor reference sequence (bottom). Mos1 5′ Inverted Terminal Repeats (5′ITR) are indicated with red arrows. See Figure 2D for overview. (PDF) [file pone.0093076.s004.pdf]

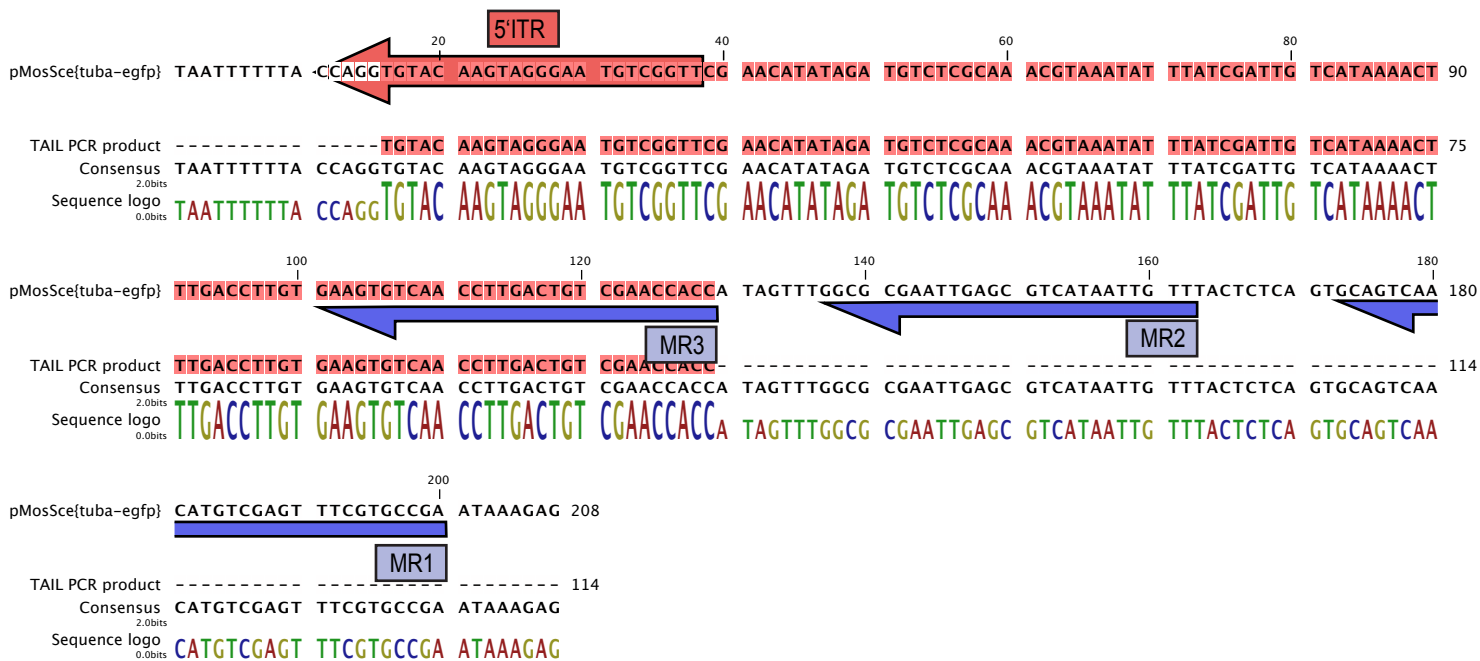

Alignment S3

Supplement: Alignment S3 — TAIL-PCR amplicon recovered from stable tuba::egfpvbci1 animals (5′ arm). Multiple sequence alignment showing the sequence of a fragment recovered by TAIL-PCR from the stable tuba::egfpvbci1 strain. The Mos1 5′ITR is indicated by a red arrow; blue arrows demarcate position of TAIL-PCR primers. (PDF) [file pone.0093076.s005.pdf]

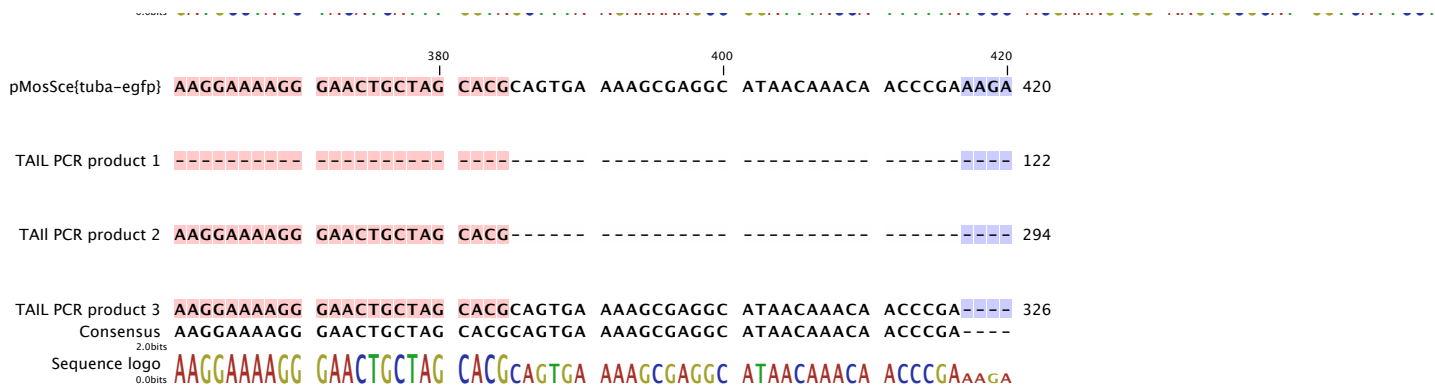

Supplement: Alignment S4 — TAIL-PCR amplicons recovered from stable tuba::egfpvbci1 animals (3′ arm). Multiple sequence alignment showing the sequences of three fragment recovered by TAIL-PCR from the stable tuba::egfpvbci1 strain. The Mos1 3′ITR is indicated by a red arrow; blue arrows demarcate position of TAIL-PCR primers as well as the gDf primer used to independently confirm the recovered integration; yellow box indicates Platynereis genomic DNA at transition between reporter and genomic DNA. (PDF) [file pone.0093076.s006.pdf]

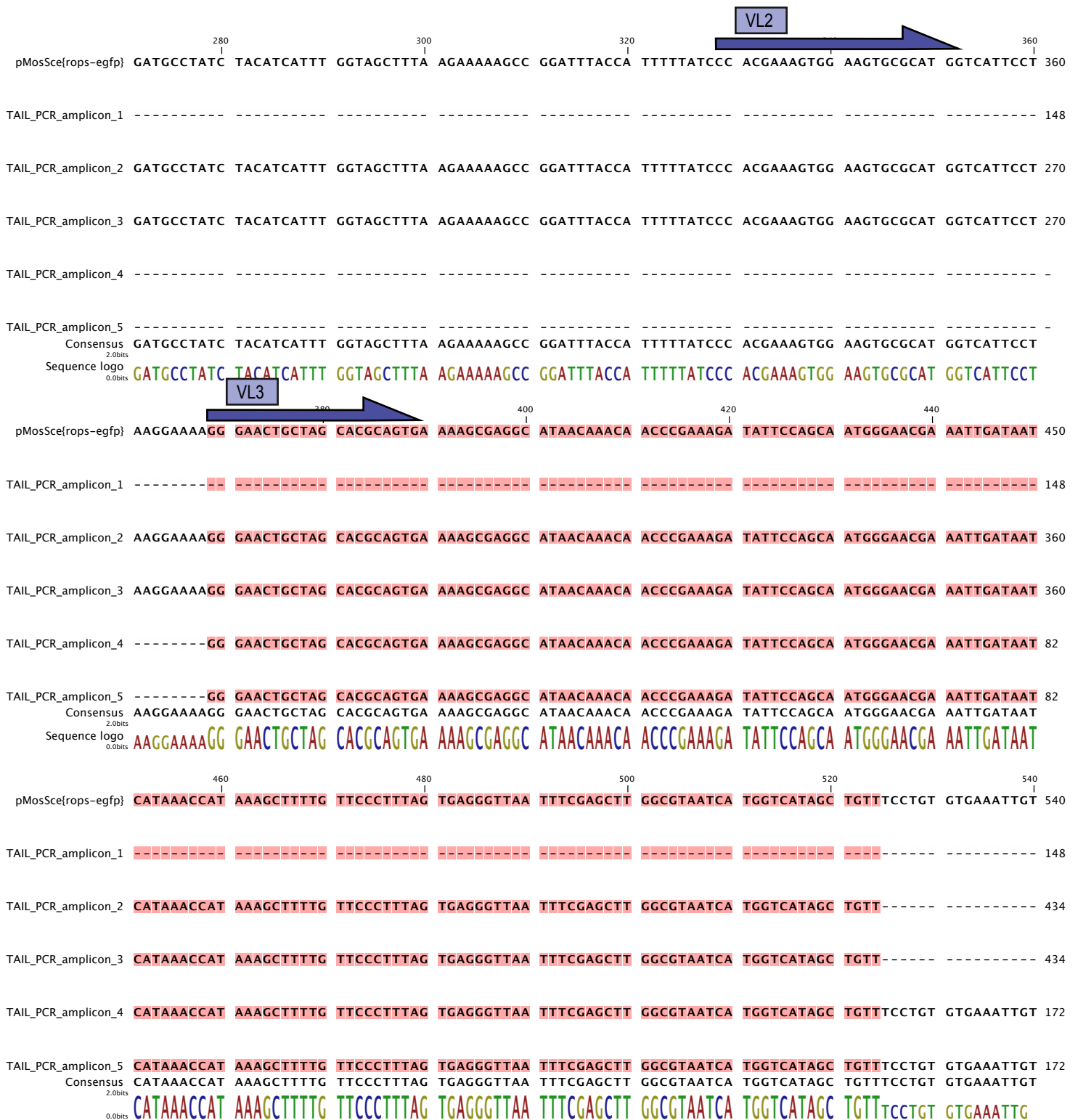

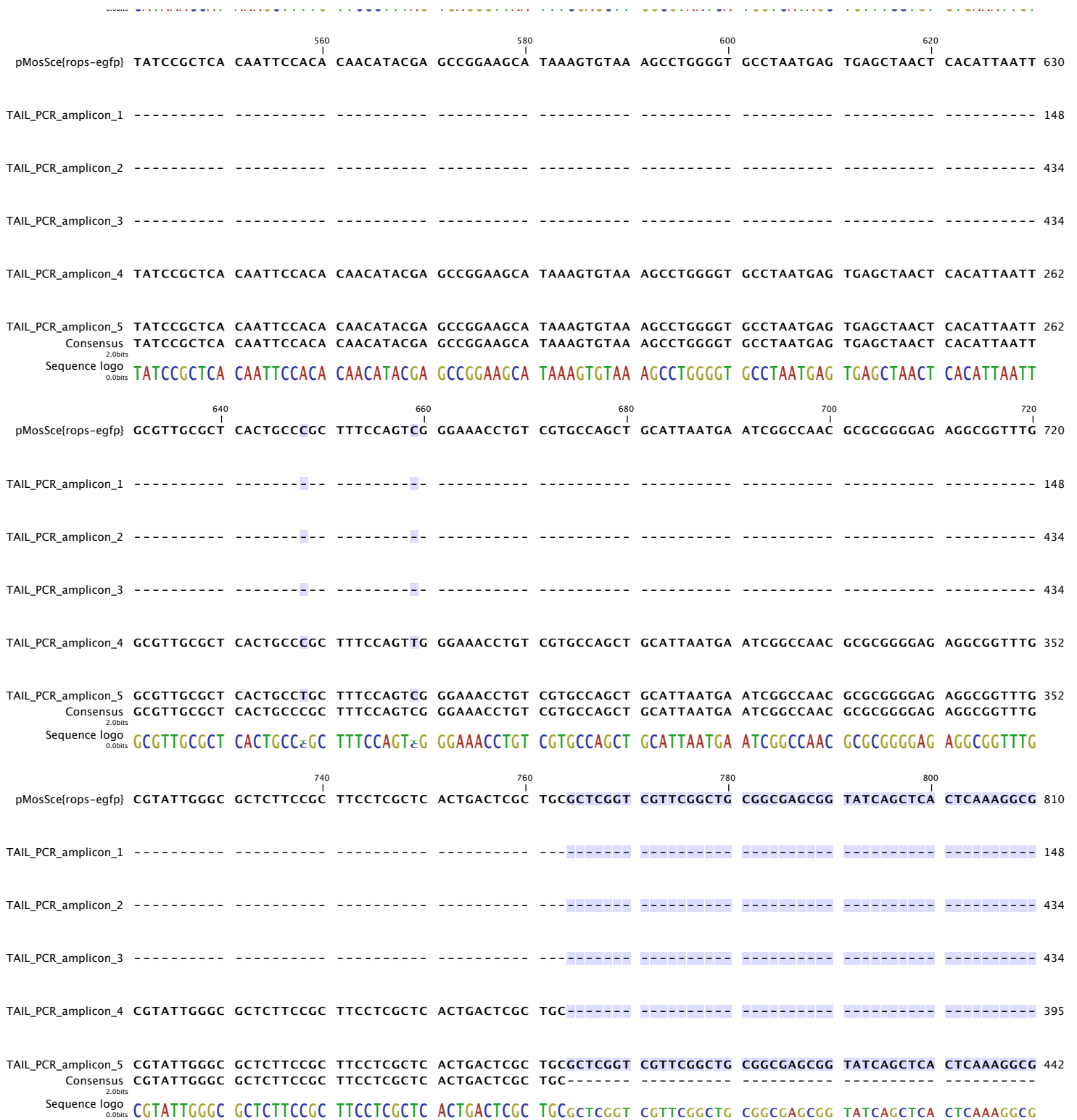

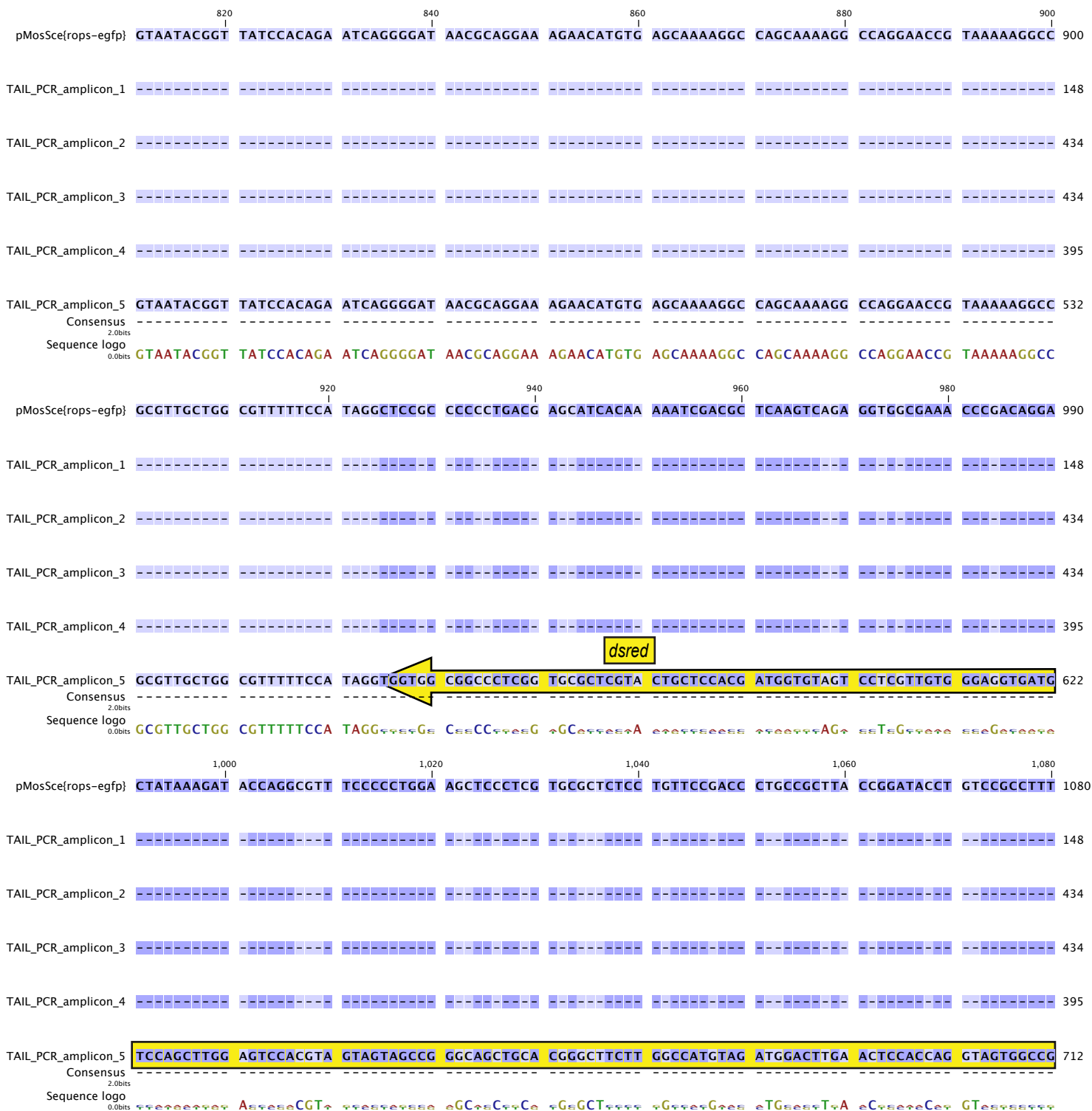

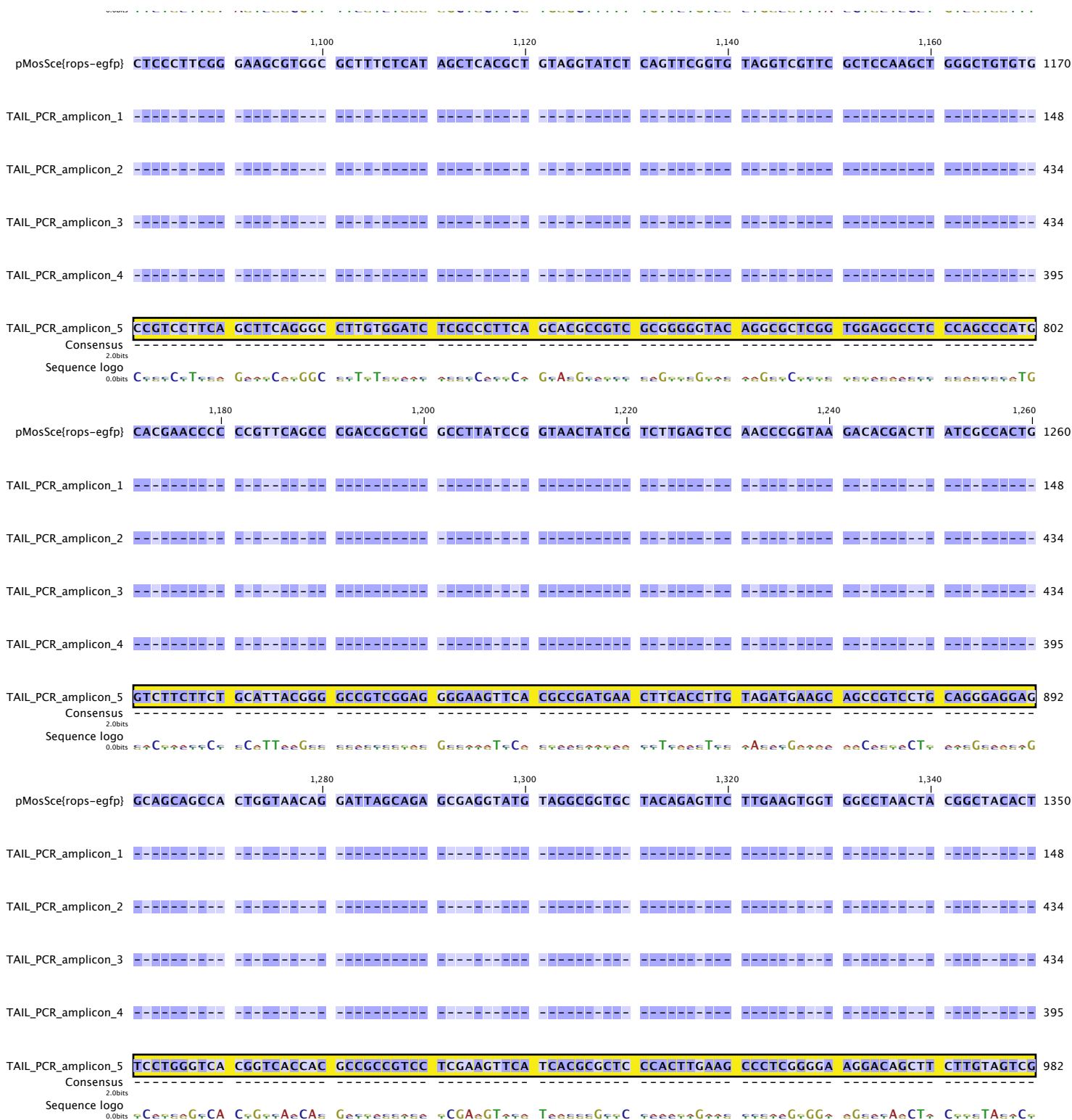

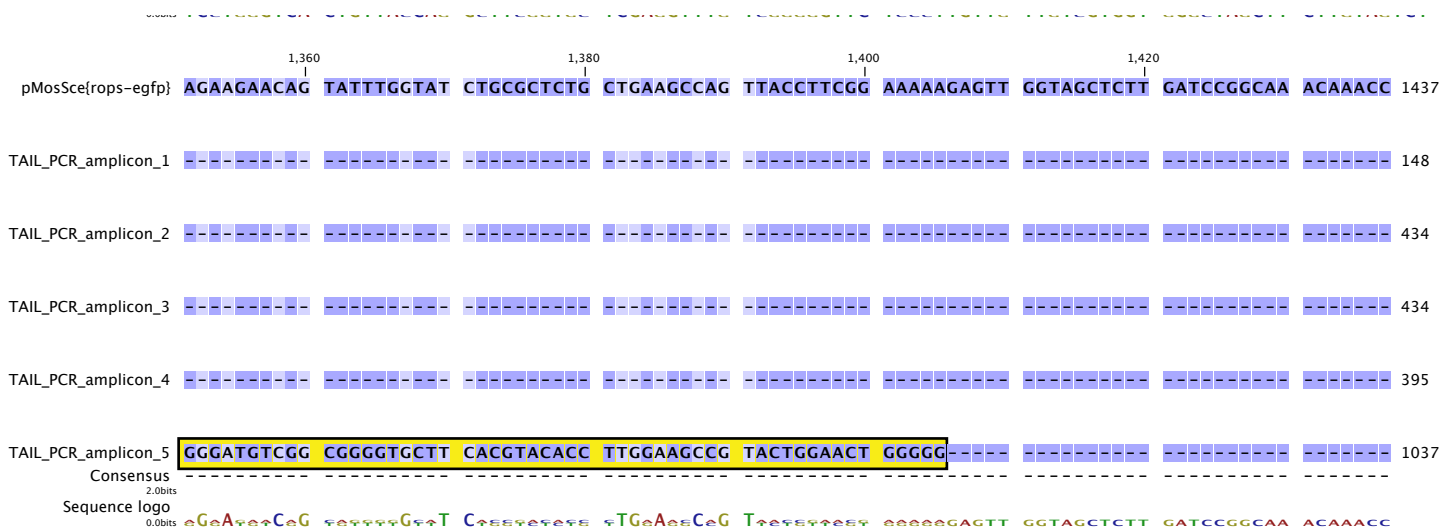

Supplement: Alignment S5 — TAIL-PCR amplicons recovered from stable rops::egfpvbci2 animals (3′ arm). Multiple sequence alignment showing the sequences of five fragments recovered by TAIL-PCR from the stable rops::egfpvbci2 strain. The Mos1 3′ITR is indicated by a red arrow; blue arrows demarcate position of TAIL-PCR primers (two sets were used); yellow box indicates dsred cDNA that it present at a different region of the donor plasmid, indicative of fragmentation of the donor prior to integration. (PDF) [file pone.0093076.s007.pdf]
