# Supplementary material for: Tools for Gene-Regulatory Analyses in the Marine Annelid Platynereis dumerilii
Source: PLoS One. 2014 Apr 8;9(4):e93076. doi: 10.1371/journal.pone.0093076 (PMC3979674; doi:10.1371/journal.pone.0093076)
Supplement: Table S1 — Sequences of primers used in this study. The table shows the sequences for all primers used in this study, along with the primer names. (DOCX) [file pone.0093076.s002.docx]

**Table S1. Sequences of primers used in this study.**

| Name | Sequence |
| --- | --- |
| rF | 5'-TAGGGATAACAGGGTAATTTCCCAGAAAAACGCTGATGTATGG-3' |
| rL | 5'-CTCCTCGCCCTTGCTCACCATCTGAAATGTTAATAATAGATAAATAAAAACATTG-3' |
| gF | 5'-GGATCCACCGGTCGCCAC-3' |
| gF2 | 5’-ATGGTGAGCAAGGGCGAGGAGC-3’ |
| gL | 5'-TAGGGATAACAGGGTAATGCTTAAGATACATTGATGAGTTTGGAC-3' |
| tF | 5'-TAGGGATAACAGGGTAATCAATTCGTCTGCCACTCTGCAAC-3' |
| tL | 5'-GTGGCGACCGGTGGATCCTGTCCGACGTGGATGCTG-3' |
| mF | 5’-TAGGGATAACAGGGTAATGGGTGGCTTTAGAAGTATGTCTG-3' |
| mL | 5’-GCTCCTCGCCCTTGCTCACCATAAGCCTGTTCTAAATATAGCTCCG-3’ |
| T2f | GCCAAGCGCGCAATTAACCCTC |
| T2r | GTACGCCTACCTGTTGGACCCAGG |
| M1f | CCCTCACTAAAGGGAACAAAAGCTTTATGG |
| M1r | CTTGCATGCTCCGCTGTTTTGTCAGTG |
| M1nf | TGACCATGCGCACTTCCACTTTCGTG |
| M1nr | GAGCGAACGCAGACGAGTAAAACG |
| RT1 | 5'-GCTTCAACTCCTGATCCAAACGCTC-3' |
| RT2 | 5'-GCGCCGGGGAGTTGTGTAGG-3' |
| RT3 | 5'-TGGAGTACACCAATGGTATGCGCG-3' |
| TL1 | 5'-CTAAAGCAGGATAAAACCTTGTATGCATTTC-3' |
| TL2 | 5'-CCTTAATACTCAAGTACAATTTTAATGGAGTAC-3' |
| TL3 | 5'-CTTTTACTCAAGTAAGATTCTAGCCAGATAC-3' |
| TR1 | 5'-CGTCACTTCCAAAGGACCAATGAACATGTC-3' |
| TR2 | 5'-CTGGGCATCAGCGCAATTCAATTGG-3' |
| TR3 | 5'-GCAAGGGAAAATAGAATGAAGTGATCTCC-3' |
| MR1 | 5'-GGCACGAAACTCGACATGTTGACTGC-3' |
| MR2 | 5'-AACAATTATGACGCTCAATTCGCGCC-3' |
| MR3 | 5'-GGTGGTTCGACAGTCAAGGTTGACACTTC-3' |
| ML1 | 5'-AGCGCTTCGATTCTTACGAAAGTGTG-3' |
| ML2 | 5'-GGTTCGCCGCAAAAGACGATGAGTTC-3' |
| ML3 | 5'-GGAATCCACAAATTGCCCGAGAGATGG-3' |
| VL1 | 5'-CACCGACTGGAGCCCGTACTGTG-3' |
| VL2 | 5'-CCACGAAAGTGGAAGTGCGCATG-3' |
| VL3 | 5'-GGGAACTGCTAGCACGCAGTG-3' |
| gDf | 5'-CAAGTCAGCCAACCAACAAATTATG-3' |
