# Supplementary material for: Tools for Gene-Regulatory Analyses in the Marine Annelid Platynereis dumerilii
Source: PLoS One. 2014 Apr 8;9(4):e93076. doi: 10.1371/journal.pone.0093076 (PMC3979674; doi:10.1371/journal.pone.0093076)

		20		40		60		80		
3_5.3-1_M13	TGACCATGCG	CACTTCCACT	TTCGTGGGAT	AAAAATGGTA	AATCCGGCTT	TTTCTTAAAG	CTACCAAATG	ATGTAGATAG	GCATCCCACA	90
13_14-1_M13	TGACCATGCG	CACTTCCACT	TTCGTGGGAT	AAAAATGGGA	AAT-------	----------	----------	----------	----------	43
6_7.3-1_M13	TGACCATGCG	CACTTCCACT	TTCGTGGGAT	AAAAATGGTA	AATCCGGCTT	TTTCTTAAAG	CTACCAAATG	ATGTAGATAG	GCATCCCACA	90
14_15.2-2_M13	TGACCATGCG	CACTTCCACT	TTCGTGGGAT	AAAAATGGTA	AATCCGGCTT	TTTCTTATAA	----------	----------	----------	60
7_12.2-2_M13	TGACCATGCG	CACTTCCACT	TTCGTGGGAT	AAAAAT----	----------	----------	----------	----------	----------	36
18_19.2_2255	TGACCATGCG	CACTTCCACT	TTCGTGGGAT	AAAAATGGTA	AATCCGGCTT	TTTCTTAAAG	CTACCAAATG	ATGTAGATAG	GCATCCCACA	90
13_16.1_2255	TGACCATGCG	CACTTCCACT	TTCGTGGGAT	AAAAATGGTA	AATCCGGCTT	TTTCTTAAAG	CTACCAAATG	ATGTAGATAG	GCATCCCACA	90
15_17.1_2255	TGACCATGCG	CACTTCCACT	TTCGTGGGAT	AAAAATGGTA	AATCCGGCTT	TTTCTTAAAG	CTACCAAATG	ATGTAGATAG	GCATCCCACA	90
11_12.2-1_M13	TGACCATGCG	CACTTCCACT	TTCGTGGGAT	AAAAATGGTA	AATCCGGCTT	TTTCTTAAAG	CTACCAAATG	ATGTAGATAG	GCATCCCACA	90
8_20.1-1_M13	TGACCATGCG	CACTTCCACT	TTCGTGGGAT	AAAAATGGTA	AATCCGGCTT	TTTCTTAAAG	CTACCAAATG	ATGTAGATAG	GCATCCCACA	90
8_5.4-1_M13	TGACCATGCG	CACTTCCACT	TTCGTGGGAT	AAAAATTCGA	AA--------	----------	----------	----------	----------	42
9_6.5-4_M13	TGACCATGCG	CACTTCCACT	TTCGTGGGAT	AAAAATGGTA	AATCCGGCTT	TTTCTTAAAG	CTACCAAATG	ATGTAGATAG	GCATC-----	85
7_5.1-4_M13	TGACCATGCG	CACTTCCACT	TTCGTGGGAT	AAAAATGGTA	AATCCGGCTT	TTTCTTAAAG	CTACCAAATG	ATGTAGATAG	GCATCCCACA	90
5_6.5-3_M13	TGACCATGCG	CACTTCCACT	TTCGTGGGAT	AAAAATGGTA	AATCCGGCTT	TTTCTTAAAG	CTACCAAATG	ATGTAGATAG	GCATCCCACA	90
10_7.2-2_M13	TGACCATGCG	CACTTCCACT	TTCGTGGGAT	AAAAATGGTA	AATCCGGCTT	TTTCTTAAAG	CTACCAAATG	ATGTAGATAG	GCATCCCACA	90
4_6.4-3_M13	TGACCATGCG	CACTTCCACT	TTCGTGGGAT	AAAAATGGTA	AATCCGGCTT	TTCCTTAAAG	CTACCAAATG	ATGTAGATAG	GCATCCCACA	90
2_5.1-2_M13	TGACCATGCG	CACTTCCACT	TTCGTGGGAT	AAAAATGGTA	AATCCGGCTT	TTTCTTAAAG	CTACCAAATG	ATGTAGATAG	GCATCCCACA	90
1_2_2255	TGACCATGCG	CACTTCCACT	TTCGTGGGAT	AAAAATGGTA	AATCCGGCTT	TTTCTTAAAG	CTACCAAATG	ATGTAGATAG	GCATCCCACA	90
7_7.2_2255	TGACCATGCG	CACTTCCACT	TTCGTGGGAT	AAAAATGGTA	AATCCGGCTT	TTTCTTAAAG	CTACCAAATG	ATGTAGATAG	GCATCCCACA	90
6_4-3_M13	TGACCATGCG	CACTTCCACT	TTCGTGGGAT	AAAAATGGTA	AATCCGGCTT	TTTCTTAAAG	CTACCAAATG	ATGTAGATAG	GCATCCCACA	90
1_4-2_M13	TGACCATGCG	CACTTCCACT	TTCGTGGGAT	AAAAATGGTA	AATCCGGCTT	TTTCTTAAAG	CTACCAAATG	ATGTAGA---	----------	77
reference	TGACCATGCG	CACTTCCACT	TTCGTGGGAT	AAAAATGGTA	AATCCGGCTT	TTTCTTAAAG	CTACCAAATG	ATGTAGATAG	GCATCCCACA	90
Consensus	TGACCATGCG	CACTTCCACT	TTCGTGGGAT	AAAAATGGTA	AATCCGGCTT	TTTCTTAAAG	CTACCAAATG	ATGTAGATAG	GCATCCCACA	

Sequence logo 2.0bits 0.0bits

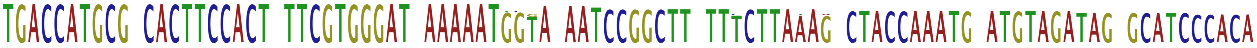

Alignment S1

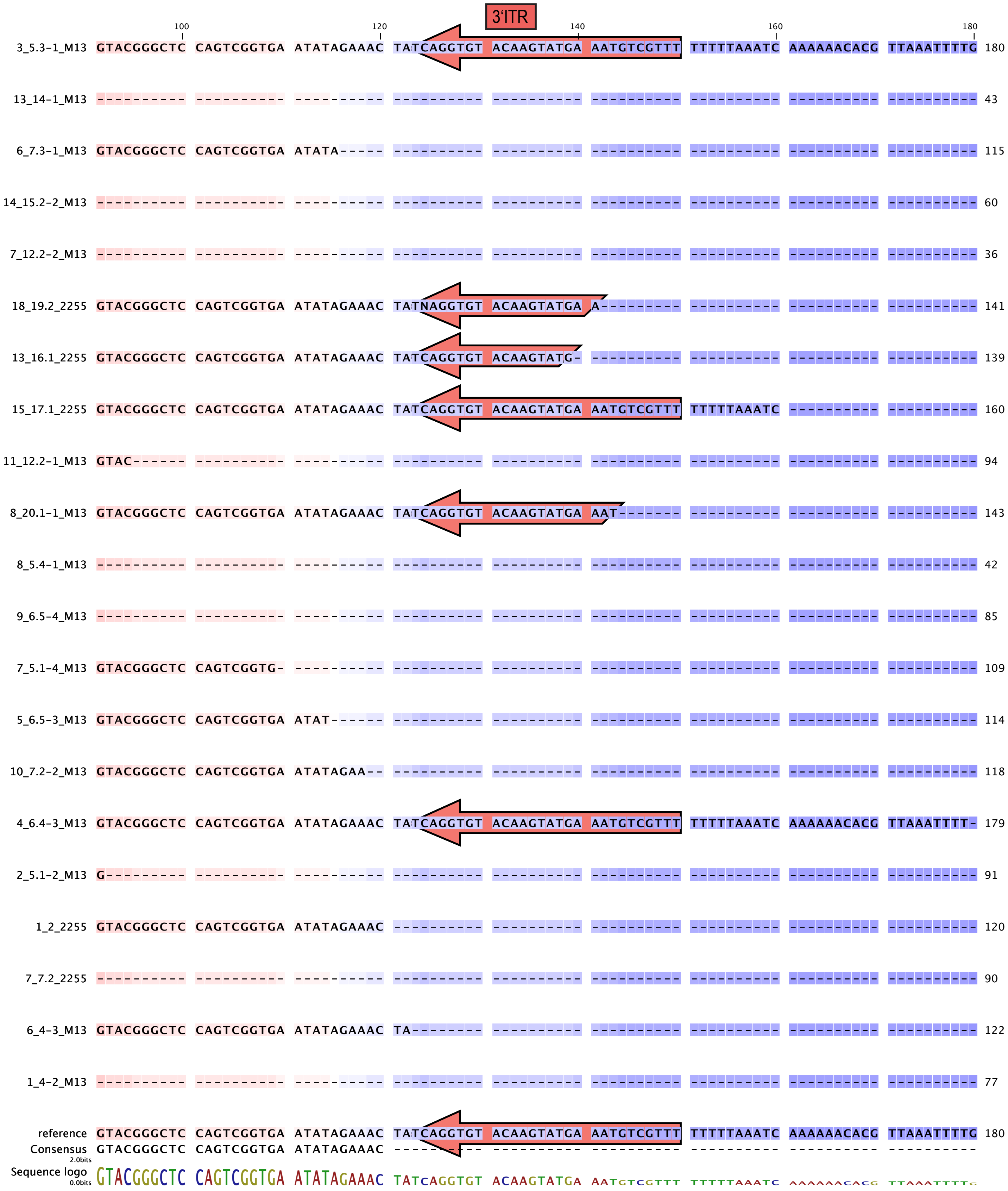
3'ITR
100 120 140 160 180
3_5.3-1_M13 GTACGGGCTC CAGTCGGTGA ATATAGAAAC TATCAGGTGT ACAAGTATGA AATGTCGTTT TTTTTAAATC AAAAAACACG TTAAATTTTG 180
13_14-1_M13 43
6_7.3-1_M13 GTACGGGCTC CAGTCGGTGA ATATA 115
14_15.2-2_M13 60
7_12.2-2_M13 36
18_19.2_2255 GTACGGGCTC CAGTCGGTGA ATATAGAAAC TATNAGGTGT ACAAGTATGA A 141
13_16.1_2255 GTACGGGCTC CAGTCGGTGA ATATAGAAAC TATCAGGTGT ACAAGTATG 139
15_17.1_2255 GTACGGGCTC CAGTCGGTGA ATATAGAAAC TATCAGGTGT ACAAGTATGA AATGTCGTTT TTTTTAAATC 160
11_12.2-1_M13 GTAC 94
8_20.1-1_M13 GTACGGGCTC CAGTCGGTGA ATATAGAAAC TATCAGGTGT ACAAGTATGA AAT 143
8_5.4-1_M13 42
9_6.5-4_M13 85
7_5.1-4_M13 GTACGGGCTC CAGTCGGTG 109
5_6.5-3_M13 GTACGGGCTC CAGTCGGTGA ATAT 114
10_7.2-2_M13 GTACGGGCTC CAGTCGGTGA ATATAGAA 118
4_6.4-3_M13 GTACGGGCTC CAGTCGGTGA ATATAGAAAC TATCAGGTGT ACAAGTATGA AATGTCGTTT TTTTTAAATC AAAAAACACG TTAAATTTT 179
2_5.1-2_M13 G 91
1_2_2255 GTACGGGCTC CAGTCGGTGA ATATAGAAAC 120
7_7.2_2255 90
6_4-3_M13 GTACGGGCTC CAGTCGGTGA ATATAGAAAC TA 122
1_4-2_M13 77
reference GTACGGGCTC CAGTCGGTGA ATATAGAAAC TATCAGGTGT ACAAGTATGA AATGTCGTTT TTTTTAAATC AAAAAACACG TTAAATTTTG 180
Consensus GTACGGGCTC CAGTCGGTGA ATATAGAAAC
Sequence logo 2.0bits 0.0bits GTACGGGCTC CAGTCGGTGA ATATAGAAAC TATCAGGTGT ACAAGTATGA AATGTCGTTT TTTTTAAATC AAAAAACACG TTAAATTTTG

		200		220			240		260	
3_5.3-1_M13	TGGAAAAAGA	AAAAATCATT	TATTCAAAGT	ATTTGCCGTC	GCTAGCTACA	CATTTTTCCC	ATCTCTCGGG	CAATTTGTGG	ATTCCACGCC	270
13_14-1_M13	----------	----------	----------	----------	----------	----------	----------	----------	----------	43
6_7.3-1_M13	----------	----------	----------	----------	----------	----------	----------	----------	----------	115
14_15.2-2_M13	----------	----------	----------	----------	----------	----------	----------	----------	----------	60
7_12.2-2_M13	----------	----------	----------	----------	----------	----------	----------	----------	----------	36
18_19.2_2255	----------	----------	----------	----------	----------	----------	----------	----------	----------	141
13_16.1_2255	----------	----------	----------	----------	----------	----------	----------	----------	----------	139
15_17.1_2255	----------	----------	----------	----------	----------	----------	----------	----------	----------	160
11_12.2-1_M13	----------	----------	----------	----------	----------	----------	----------	----------	----------	94
8_20.1-1_M13	----------	----------	----------	----------	----------	----------	----------	----------	----------	143
8_5.4-1_M13	----------	----------	----------	----------	----------	----------	----------	----------	----------	42
9_6.5-4_M13	----------	----------	----------	----------	----------	----------	----------	----------	----------	85
7_5.1-4_M13	----------	----------	----------	----------	----------	----------	----------	----------	----------	109
5_6.5-3_M13	----------	----------	----------	----------	----------	----------	----------	----------	----------	114
10_7.2-2_M13	----------	----------	----------	----------	----------	----------	----------	----------	----------	118
4_6.4-3_M13	----------	----------	----------	----------	----------	----------	----------	----------	----------	179
2_5.1-2_M13	----------	----------	----------	----------	----------	----------	----------	----------	----------	91
1_2_2255	----------	----------	----------	----------	----------	----------	----------	----------	----------	120
7_7.2_2255	----------	----------	----------	----------	----------	----------	----------	----------	----------	90
6_4-3_M13	----------	----------	----------	----------	----------	----------	----------	----------	----------	122
1_4-2_M13	----------	----------	----------	----------	----------	----------	----------	----------	----------	77
reference	TGGAAAAAGA	AAAAATCATT	TATTCAAAGT	ATTTGCCGTC	GCTAGCTACA	CATTTTTCCC	ATCTCTCGGG	CAATTTGTGG	ATTCCACGCC	270
Consensus	----------	----------	----------	----------	----------	----------	----------	----------	----------	
Sequence logo (2.0bits / 0.0bits)										

	280		300		320	
3_5.3-1_M13	AGTAGAACTC	ATCGTCTTTT	GCGGCGAACC	ATTCATCGAG	CCATTT----	316
13_14-1_M13	----------	----------	----------	----------	----------	43
6_7.3-1_M13	----------	----------	----------	----------	----------	115
14_15.2-2_M13	----------	----------	----------	----------	----------	60
7_12.2-2_M13	----------	----------	----------	----------	----------	36
18_19.2_2255	----------	----------	----------	----------	----------	141
13_16.1_2255	----------	----------	----------	----------	----------	139
15_17.1_2255	----------	----------	----------	----------	----------	160
11_12.2-1_M13	----------	----------	----------	----------	----------	94
8_20.1-1_M13	----------	----------	----------	----------	----------	143
8_5.4-1_M13	----------	----------	----------	----------	----------	42
9_6.5-4_M13	----------	----------	----------	----------	----------	85
7_5.1-4_M13	----------	----------	----------	----------	----------	109
5_6.5-3_M13	----------	----------	----------	----------	----------	114
10_7.2-2_M13	----------	----------	----------	----------	----------	118
4_6.4-3_M13	----------	----------	----------	----------	----------	179
2_5.1-2_M13	----------	----------	----------	----------	----------	91
1_2_2255	----------	----------	----------	----------	----------	120
7_7.2_2255	----------	----------	----------	----------	----------	90
6_4-3_M13	----------	----------	----------	----------	----------	122
1_4-2_M13	----------	----------	----------	----------	----------	77
reference	AGTAGAACTC	ATCGTCTTTT	GCGGCGAACC	ATTCATCGAG	CCATTTTTTC	320
Consensus	----------	----------	----------	----------	----------	

Sequence logo

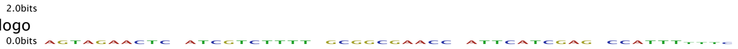

Supplement: Alignment S1 — Donor sequences recovered from mos1 -injected worms (3′ arm). Multiple sequence alignment showing sequences of fragments recovered from the excision assay in comparison to the pMos{rps9::egfp}frkt1074 donor reference sequence (bottom). Mos1 3′ Inverted Terminal Repeats (3′ITR) are indicated with red arrows; see Figure 2D for overview. The alignment does not show the following insertions that lie between the displayed regions of the amplicons and the regions in Alignment S2 and cannot be aligned to the reference sequence: 13_14-1_M13: GTCCCTT (7 bp); 7_12.2-2_M13: TGGA (4 bp); 11_12.2-1_M13: AACCCGGAATGACCATGCGCATCC (24 bp); 8_20.1-1_M13: AA (2 bp); 9_6.5-4_M13: A (1 bp); 5_6.5-3_M13: GGCTCC (6 bp); 10_7.2-2_M13: TAATTAGACAAAGTGAAG (18 bp). (PDF) [file pone.0093076.s003.pdf]
